# Subtracting the sequence bias from partially digested MNase-seq data reveals a general contribution of TFIIS to nucleosome positioning

**DOI:** 10.1186/s13072-017-0165-x

**Published:** 2017-12-07

**Authors:** Gabriel Gutiérrez, Gonzalo Millán-Zambrano, Daniel A. Medina, Antonio Jordán-Pla, José E. Pérez-Ortín, Xenia Peñate, Sebastián Chávez

**Affiliations:** 1 Departamento de Genética, Universidad de Sevilla, Seville, Spain; 2 Instituto de Biomedicina de Sevilla, Universidad de Sevilla-CSIC-Hospital Universitario V. del Rocío, Seville, Spain; 3 Present Address: The Gurdon Institute, University of Cambridge, Cambridge, UK; 4 Departamento de Bioquímica y Biología Molecular, Universitat de València, Burjassot, Valencia, Spain; 5 Present Address: Department of Chemical and Bioprocess Engineering, Pontificia Universidad Católica de Chile, Santiago, Chile; 6 Present Address: Department of Molecular Biosciences, The Wenner-Gren Institute, Stockholm University, Stockholm, Sweden

**Keywords:** Nucleosome mapping, MNase-seq, MNase-sensitive nucleosomes, TFIIS, Nucleosomal fuzziness

## Abstract

**Background:**

TFIIS stimulates RNA cleavage by RNA polymerase II and promotes the resolution of backtracking events. TFIIS acts in the chromatin context, but its contribution to the chromatin landscape has not yet been investigated. Co-transcriptional chromatin alterations include subtle changes in nucleosome positioning, like those expected to be elicited by TFIIS, which are elusive to detect. The most popular method to map nucleosomes involves intensive chromatin digestion by micrococcal nuclease (MNase). Maps based on these exhaustively digested samples miss any MNase-sensitive nucleosomes caused by transcription. In contrast, partial digestion approaches preserve such nucleosomes, but introduce noise due to MNase sequence preferences. A systematic way of correcting this bias for massively parallel sequencing experiments is still missing.

**Results:**

To investigate the contribution of TFIIS to the chromatin landscape, we developed a refined nucleosome-mapping method in *Saccharomyces cerevisiae*. Based on partial MNase digestion and a sequence-bias correction derived from naked DNA cleavage, the refined method efficiently mapped nucleosomes in promoter regions rich in MNase-sensitive structures. The naked DNA correction was also important for mapping gene body nucleosomes, particularly in those genes whose core promoters contain a canonical TATA element. With this improved method, we analyzed the global nucleosomal changes caused by lack of TFIIS. We detected a general increase in nucleosomal fuzziness and more restricted changes in nucleosome occupancy, which concentrated in some gene categories. The TATA-containing genes were preferentially associated with decreased occupancy in gene bodies, whereas the TATA-like genes did so with increased fuzziness. The detected chromatin alterations correlated with functional defects in nascent transcription, as revealed by genomic run-on experiments.

**Conclusions:**

The combination of partial MNase digestion and naked DNA correction of the sequence bias is a precise nucleosomal mapping method that does not exclude MNase-sensitive nucleosomes. This method is useful for detecting subtle alterations in nucleosome positioning produced by lack of TFIIS. Their analysis revealed that TFIIS generally contributed to nucleosome positioning in both gene promoters and bodies. The independent effect of lack of TFIIS on nucleosome occupancy and fuzziness supports the existence of alternative chromatin dynamics during transcription elongation.

**Electronic supplementary material:**

The online version of this article (10.1186/s13072-017-0165-x) contains supplementary material, which is available to authorized users.

## Background

DNA is compacted into nucleosomes in all eukaryotes [1]. Nucleosomes are histone octamers (two copies of each histone: H2A, H2B, H3, and H4) surrounded by approximately 1.7 DNA turns [2, 3]. In the long DNA molecule that constitutes a chromosome, nucleosomes are arranged in a beads-on-a-string way and are the basic elements that contribute to form all the higher-order structures that constitute chromatin. Chromatin is highly dynamic and influences all the fundamental processes that take place in DNA, e.g., replication, repair, recombination and transcription.

It is increasingly clear that nucleosomes inhibit transcription elongation [4, 5]. Conversely, transcription shapes nucleosome positioning, i.e., the exact location of nucleosomes in relation to the DNA sequence across the genome [6, 7]. TFIIS is a transcription elongation factor required to solve backtracking, a situation in which RNA polymerase II slides back over the template DNA and the active site is separated from the 3′ end of the nascent transcript [8]. Backtracked polymerase cannot elongate until an RNA-cleavage reaction takes place, which generates a new 3′ end at the active site. This reaction is strongly enhanced by the interaction of TFIIS with the active RNA polymerase II site [9]. TFIIS is also known to help transcription through chromatin in vitro [10]. Accordingly, and given the bidirectional relationship between transcription and nucleosome positioning, RNA polymerase II backtracking and its resolution by TFIIS could influence nucleosome positioning across the genome. Yet as far as we know, no evidence for this has been found to date.

One of the most popular tools to study nucleosome positioning involves the digestion of chromatin samples with micrococcal nuclease (MNase) since this enzyme preferentially cuts internucleosomal (linker) DNA [11]. Thus, fixing cells with formaldehyde and digesting chromatin with MNase generate a collection of DNA fragments that contains information about the position that each nucleosome occupies in DNA. This technique is easy and has proved useful for several different organisms [12–15]. Nevertheless, two important drawbacks need to be taken into account: MNase has a certain sequence preference [16, 17], and this enzyme can also digest nucleosomal DNA if no more internucleosomal DNA is left (overdigestion) [12, 17]. This last property of MNase has led some researchers to conduct partial digestion which, in turn, enhances the sequence bias. Correcting this bias has always been a challenge. Classical experiments include a control in which naked DNA is digested and the resulting pattern is compared to partially digested chromatin samples [18–20]. This is possible because only a relatively short region of the genome is analyzed in these experiments. More recently, however, deep sequencing technology has been used to study nucleosome positioning at the genome-wide level (MNase-seq) [12, 21–23]. Nucleosomal maps based on intensively digested chromatin samples miss the so-called fragile (MNase-sensitive) nucleosomes [12, 24, 25], and those that rely on gently digested chromatin introduce noise due to MNase sequence preference. A trustworthy method for sequence-bias correction in these experiments is still missing.

We herein propose a correction procedure based on actually measuring naked DNA cleavage and using a bioinformatics tool to subtract the naked DNA signal from the chromatin data in partially digested MNase-seq experiments. We show that the combination of gentle MNase digestion (not overdigested) with the naked DNA correction improves the quality of the MNase-seq data and can be used in a wide range of experiments from now on. By using this improved method, we found differences between samples that were hidden behind the noise generated by the sequence bias. We found that lack of TFIIS influences nucleosome occupancy across the genome in both gene bodies and promoter regions. This influence was particularly strong in the genes that contained a canonical TATA box (TATA genes), which were also more drastically affected by the sequence-bias correction. This effect on nucleosome occupancy was related with the transcriptional function of TFIIS since the nucleosome profiles of those genes whose transcription rate was more drastically affected by lack of TFIIS exhibited strong alterations. The improved method was particularly useful for accurately measuring changes in nucleosomal fuzziness independently of occupancy alterations, which led us to detect a very general contribution of TFIIS to nucleosome positioning along the gene body.

## Methods

### Yeast strains and media

Yeast strain BY4741 (MATa, *his3Δ1*, *leu2Δ0*, *met15Δ0*, *ura3Δ0*) was used as the wild type (wt). *dst1∆* is a BY4741 derivative. All the yeast strains used in Additional file 1 (wt, *rsc9ts*, *set1∆* and *rsc9ts set1∆*) were derived from W303 (MATa, *ade2*-*1*, *can1*-*100*, *his3*-*11*, *leu2*-*3*, *ura3*-*1*). Strains were grown in YPD (1% yeast extract, 2% peptone and 2% glucose).

### MNase digestion

The MNase digestion protocol was derived from [26] with modifications detailed herein. First 500 ml of exponentially grown yeast culture was cross-linked by adding formaldehyde until a final concentration of 1%, followed by 15-min incubation at room temperature. For quenching, glycine was added to 2% and the sample was incubated for 5 min at room temperature. Cells were washed 3 times with cold distilled water and were resuspended in 10 ml of Buffer Z2 (1 M Sorbitol; 50 mM Tris-HCl pH 7.4; 10 mM β-mercaptoethanol). Spheroplasts were prepared by adding 50 μl of Zymolyase 100T (20 μg/ml). Samples were incubated at 30 °C for approximately 30 min until the optical density at 600 nm was around 10% of the initial value. After washing spheroplasts twice with cold washing buffer I (1 M sorbitol; 20 mM Tris-HCl, pH 8; 1 mM EDTA; 150 mM NaCl), they were resuspended in 1.5 ml of buffer II (20 mM Tris-HCl, pH 8; 1 mM EDTA; 150 mM NaCl; 0.1 mM PMSF; 0.2% Triton X-100). Spheroplasts were separated into five tubes with 3 ml of MNase buffer (15 mM Tris-HCl, pH 8; 50 mM NaCl; 1.4 mM CaCl2; 0.2 mM EGTA; 0.2 mM EDTA) and into different amounts of micrococcal nuclease, which ranged from 500 to 10 mU. Samples were incubated for 30 min at 37 °C, and reactions were stopped by adding 100 μl of stop buffer (0.4 M EDTA; 0.3 M Tris-HCl, pH 8) and 150 μl of 10% SDS. Subsequently, samples were treated with 75 μl of proteinase K (20 mg/ml) for 30 min at 37 °C, and then at 65 °C for at least 1 h. After phenol/chloroform/isoamyl alcohol extraction, DNA was ethanol-precipitated, resuspended in double-distilled water and treated with 170 µg/ml of RNase A for 30 min at room temperature. Digested DNA was resolved in 2% agarose gels. One of the digestions was chosen according to the following criteria: (1) the trinucleosome band was visible, which indicates negligible overdigestion; (2) the mononucleosome band was the most intense (see Additional file 1 A). The band that corresponded to mononucleosomes was purified with the Qiagen Gel Extraction kit. DNAs were analyzed by either real-time quantitative PCR or massively parallel sequencing.

Naked DNA was prepared by treating cells exactly as before, but up to the point of RNase treatment, with the only difference being that no MNase was added. From that point onward, naked DNA was divided into four different tubes with MNase buffer and into different amounts of MNase, which ranged from 15 to 1 mU. The digestion with a similar range of fragments to that of the chosen chromatin sample (see Additional file 1 A) was electrophoresed in parallel with the chromatin sample. A slice of gel that contained these naked DNA fragments, with a similar size to the mononucleosome, was cut and DNA was purified.

### qPCR

Samples were analyzed by real-time quantitative PCR using SYBR Green Premix Ex Taq (Takara) in a Light Cycler 480 II (Roche). The reaction mixes contained 5 μl of 2X SYBR Green Premix, 4 μl of DNA and 1 μl of a primers mix (0.1 nmoles/μl each). The manufacturer’s instructions were followed.

### Sequencing and data analysis

The naked DNA sample and one *dst1∆* mutant DNA sample were used to create sequencing libraries, which were subjected to 36-nucleotide single-read sequencing in a Solexa Genome Analyzer IIx (Illumina). After quality control checking, sequencing reads were aligned to the *S. cerevisiae* reference genome (sacCer2) with the GEM software [27], which allowed up to three mismatches/reads. Only unambiguous mapped reads were used. The second *dst1∆* mutant DNA sample was subjected to 50-nucleotide single-read sequencing in an Illumina HiSeq 2000 machine. The same previous mapping procedure was performed for the second *dst1∆* sample. Our data have been uploaded to the GEO database with accession number GSE94313. The wt samples used in our analysis (GSM1018226 and GSM1018227) were taken from [28]. These samples were sequenced in the same run and under the same conditions as our naked and first mutant samples. The total genome sequencing coverage of the unambiguously mapped reads was 69X for the wt, 48X for the naked sample and 33X for the mutant one. The resulting BED files of all samples were run through the DANPOS v2.1.2 [29] algorithm, in which reads were clonally cut to remove any potential PCR amplification bias, smoothed and adjusted for nucleosome size to enhance the signal-to-noise ratio. DANPOS was run with default parameters. DANPOS pools the replica of the same condition and can use naked data as a background correction.

Metagene spectral analysis periodograms, correlation coefficients, statistical tests and heat maps of nucleosomal occupancy *versus* fuzziness were calculated with PAST, v3 [30]. In the periodograms, the power axis is expressed in proportional units to the square of the amplitudes of the sinusoids present in the data. Heat maps were computed with the kernel density option (function Gaussian, 100 × 100 columns–rows and radius 30) of PAST.

The nearest distance between the consensus centers of nucleosomes determined by us with DANPOS and the centers determined by other authors (see “Results” section and Additional file 3) was calculated with an in-house Perl script. For comparison purposes, the data about the nucleosome centers provided by [31, 12] were compiled. Then, the frequency of each distance was computed and represented in a moving-average (10 pb) histogram (Additional file 3 A). To compare the histograms, the cumulative frequency for each one was computed, and then an UPGMA tree, based on the Euclidean distance of the cumulative frequencies, was computed with PAST (Additional file 3 B).

### Genome-wide measurements of active RNA polymerases

Genomic Run-On (GRO) was done in biological triplicates by essentially following the protocol described in [32]. First, 5 × 10^8^ exponentially growing yeast cells (OD_600_ = 0.5) for the run-on reaction were used. The GRO samples provided nascent transcription rates (nTR) for all the yeast genes.

Transcriptome data were obtained from the hybridization of labeled cDNA on nylon filters [32]. The total concentration of the mRNA in yeast cells was determined by quantifying polyA + in the total RNA samples by oligo-dT hybridization of a dot-blot following the protocol described elsewhere [33] and dividing by the average cell volume.

The Bio-GRO protocol was essentially followed as described in [5, 34]. Run-on reactions were performed as previously described for GRO, but with the modifications required for biotinylated precursors. From each Bio-GRO sample, at least two biological replicates were performed. 10^9^ exponentially growing yeast cells were used (OD_600_ = 0.5). Cells were collected by centrifugation and frozen in liquid nitrogen. RNA extraction was done using the “MasterPure Yeast RNA Purification Kit” (Epicentre), following the manufacturer’s instructions. Once extracted, genomic DNA was removed by digesting with 2 µL of RNase-free DNase I (Roche) for 30 min at 37 °C. Purified RNA was resuspended in milli-Q water and spectrophotometrically quantified. Nascent RNAs (< 200 nt on average) were separated from the larger unlabeled molecules with the “miRNA NucleoSpin microRNAs isolation kit” (Macherey–Nagel) and quantified. Next, 5 µg of purified nascent RNAs was hybridized directly into a Custom Tiling Array (PN 520055, Affymetrix, Santa Clara, CA). The raw CEL images were processed with the Tiling Analysis Software (TAS, Affymetrix). The Bioconductor (http://www.bioconductor.org/packages/2.11/bioc/html/tilingArray.html) and custom R scripting packages were used for the metagene analysis. The Bio-GRO samples were normalized against the yeast genomic DNA hybridized into the same custom Affymetrix arrays.

## Results

### Improvement of nucleosome mapping by subtracting the sequence bias in gentle MNase digestion

Nucleosome-mapping procedures based on the isolation of DNA fragments from extensively MNase-digested chromatin miss information from MNase-sensitive nucleosomes. This can be avoided by performing mild MNase digestion. However, this choice is known to present two problems: (1) the analysis is limited to the mononucleosomal band, which can be depleted of some DNA segments (part of the digested DNA is left in the gel; see Additional file 1 A); (2) the potential bias introduced by the DNA sequence preference of MNase. As the first problem can be a consequence of the second one, and given the stronger misleading potential of the DNA sequence preference, we decided to focus on it. To investigate this issue, we designed and performed the experiment depicted in Additional file 1 A. *Saccharomyces cerevisiae* cells were fixed, permeabilized and digested with MNase. Nucleosome-like fragments were obtained from naked DNA digestion and from classical chromatin DNA preparation. To minimize loss of information by overdigestion, the mononucleosomal DNA from the chromatin samples, in which di- and trinucleosomal particles were still clearly visible, was gel-extracted (Additional file 1 A). To obtain a comparable MNase-treated naked DNA sample, an equivalent digestion range was chosen (see the blue arrow in Additional file 1 A) and the nucleosomal-sized fragments were gel-extracted (see the red squares in Additional file 1 A).

The recovered samples were first studied by qPCR using overlapping short amplicons. The region that contained *STL1* was analyzed. *STL1* is the gene whose nucleosomal map we previously studied in detail [35]. As expected from previous works [16, 36–38], the naked DNA pattern was not a flat line, but contained peaks and valleys (Additional file 1 B), which indicates sequence preference by MNase. The naked DNA samples obtained from different mutant strains that affected the *STL1* nucleosomal structure [39] produced the same pattern (Additional file 1 C), which confirms that this pattern is influenced exclusively by the DNA sequence. When the chromatin qPCR signal was normalized by dividing its values by those obtained from naked DNA, a clearly more defined pattern appeared: some peaks that were barely visible in the raw data became clear in the normalized data (Additional file 1 B). This result not only confirmed that the noise introduced by the MNase sequence preference interfered with the profiling of the nucleosomal landscape, as previously described [36], but also suggested that the information obtained from the naked DNA digestion could be used to improve nucleosomal mapping.

In order to extend this study genome-wide, the massively parallel sequencing of both the mononucleosomal DNA and naked DNA fragments of a nucleosomal-like size was carried out. We endeavored to find a way to use our sequenced naked DNA data to correct our chromatin data in these MNase-seq experiments. We utilized DANPOS [29], a tool developed to compare the data from different MNase-treated samples. This tool normalizes the sequencing depths from the different samples and then calculates the differential signal. Thus, when comparing our chromatin sample with our naked DNA sample, we expected to see a cleaner signal, and one free of sequence-bias noise. DANPOS identified 56,743 nucleosomes before the naked DNA correction, a number lower than previous reports [6], which could be due to the higher stringency of this software. When DANPOS corrected the raw data with the naked DNA information, as with the qPCR data, some peaks in the MNase-seq data came over more clearly (Additional file 1 D).

We wondered whether the refinement of the signal observed after correction was general or if it applied only to some particular nucleosomes. Five different pieces of evidence indicated that it was a general phenomenon. First, the total number of peaks detected by DANPOS was significantly smaller after correction (45,390 vs. 56,743 in the raw data). Certain peaks were no longer considered on the corrected map because they were found to be most prominent in the naked DNA data. We are aware that this implies simplification and that some of these peaks could still correspond to real nucleosomes, but we hypothesized that the cleaning effect of correction would exceed the drawbacks. So, the blank positions on the final map do not necessarily indicate lack of chromatin, but lack of a reliable positioned nucleosome. Accordingly, after applying a Poisson test (*p* < 0.01), there were more peaks in the corrected data than in the raw data (25,549 vs. 23,495), which supports the improved resolution of the nucleosomal map.

**Fig. 1:**
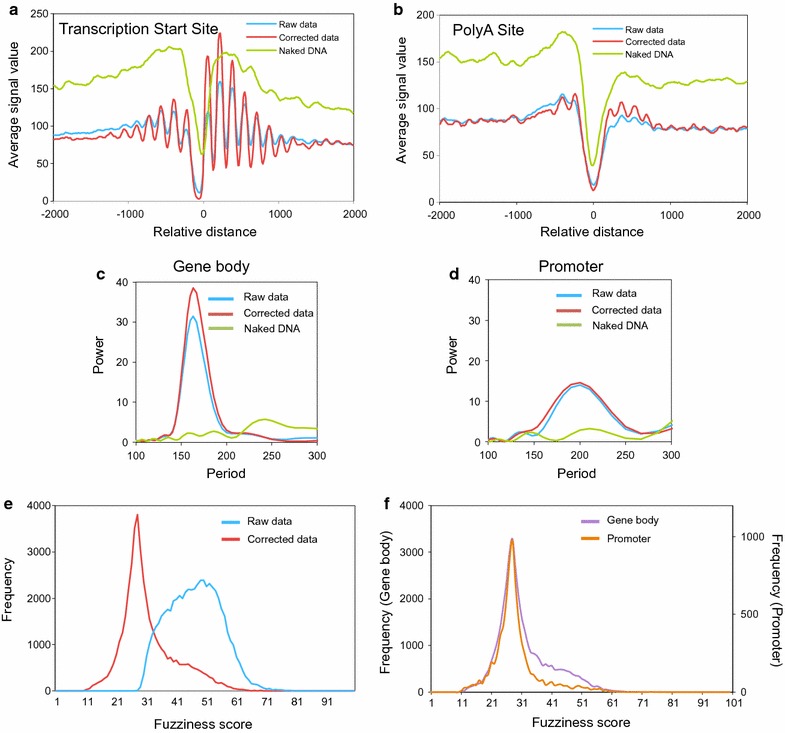
Naked DNA correction improves nucleosome mapping from partially digested MNase-seq data. **a**, **b** Metagene analysis of occupancy in the chromatin (blue before the correction, red after the correction) and naked DNA signals (green). Genes were scaled to the same length and then aligned to their TSS (**a**) or their pAS (**b**) according to the data in [66]. All the genes in the yeast genome, for which a TSS/pAS is available, were considered. **c**, **d** Spectral analysis of the promoter and gene body nucleosomes. Genes were scaled to the same length and then aligned to their TSS as **a**. A spectral analysis was plotted after classifying nucleosomes in the gene body (in the 0–1000 region) (**c**) and promoter nucleosomes (in the − 500 to 0 region) (**d**). **e** The fuzziness score distribution before (blue) and after correction (red). **f** Comparison of the fuzziness score distribution after correction, between gene bodies and promoter regions, as defined in **c**

A second piece of evidence stemmed from the data alignment in relation to the transcription start site (TSS) or the polyadenylation site (pAS). If the sequence bias of MNase was negligible, we expected to find a flat signal in the metagene analysis of the naked DNA sample, as opposed to the characteristic peaks and valleys of chromatin DNA. Interestingly, naked DNA presented a clear valley near both the TSS and pAS (Fig. 1a, b), which indicated that the DNA at these sites could have distinct physical properties, as previously suggested [38, 40]. The naked DNA correction clearly improved the nucleosomal metagene profiles. In the raw chromatin data, a nucleosome-depleted region (NDR) appeared at both these functionally relevant sites, with well-positioned nucleosomes around them, as already described [14, 41]. In the corrected data, the overall pattern was the same, but peaks were considerably larger (Fig. 1a, b). This finding agrees with a better resolution of the nucleosomal map across the genome. One difference between the TSS and pAS in this regard is that, while the position of the minimal signal in naked DNA came very close to that of chromatin in the pAS region, a shift of 50 bp took place at the TSS, with the point of the minimal signal in chromatin around position − 70, and around − 20 in naked DNA (Fig. 1a and Additional file 2 A, B). It has been shown that nucleosome positioning in pAS is influenced by the nearby TSS, which is more dependent on transcriptional activity and is determined less by intrinsic histone–DNA interactions than at the TSS [40]. We generated the metagene profiles of the 3629 genes where pAS was located at more than 500 bp from the TSS of the next gene. In agreement with [40], we found less defined nucleosomal profiles in this case (Additional file 2 C).

A third approach was to plot the log *p* value of the difference between the peaks before and after the naked DNA correction. A Poisson test was applied to test whether the corrected data differed from the raw data. To better distinguish between an increase and a decrease in the signal after corrections, we plotted − log *p* if the signal difference was positive (any raw data higher than the corrected data). We plotted log *p* if the difference was negative (any raw data lower than the corrected data). As shown in Additional file 2 C, for both the TSS and pAS alignments, the positive differences were generally more significant than the negative ones. This meant that the correction was more significant when the nucleosomal signal decreased than when it increased. However, the more regular pattern produced by the negative differences around the TSS (the red line in Additional file 2 C) indicated that those corrections which increased signals were more informative for the nucleosome positioning in these regions.

The fourth clue came from the periodicity analysis. Nucleosomal DNA is 147 bp long, and internucleosomal linker DNA is around 18 bp long in *Saccharomyces cerevisiae* [20, 42]. It is well known that nucleosomal arrays are more evenly distributed in gene bodies than in promoter regions, where they exhibit a more variable linker length and less tight DNA wrapping around the histone octamer [12]. We investigated the effect of the naked DNA correction on the nucleosomal map by applying a spectral analysis, a tool used to search for periodicity in a data set. We found that gene body nucleosomes showed a main periodicity peak at 167 bp in both the raw and corrected profiles (Fig. 1c), which agrees well with the addition of the above-mentioned nucleosome and linker lengths. Interestingly, the strength of this periodicity, measured by the Power parameter of the spectral analysis, was greater in the corrected data set. As expected, promoter nucleosomes exhibited a main periodicity peak at around 200 bp, which was clearly higher than the value obtained for the gene bodies. No effect of the naked DNA correction was observed in this case (Fig. 1d). So, the naked DNA correction improved the periodicity of those parts of the nucleosomal map that exhibited highly regular internucleosomal spacing.

Finally, we detected that the MNase sequence bias had a strong impact on nucleosome fuzziness. Most positioned nucleosomes are not in exactly the same position in each cell of the population, which makes them “fuzzy” when analyzed by MNase-seq. The distribution of the DANPOS fuzziness scores of the raw data was dome-shaped, with a mode of 47. Instead, the corrected data displayed a much steeper distribution with a mode of 28, and the two distributions significantly differed (a two-sample Kolmogorov–Smirnov test, *p* < 0.0001) (Fig. 1e). This difference meant that a substantial part of the apparent fuzziness of raw data was actually noise, due to the MNase sequence bias. This accurate measurement allowed the detection of a subpopulation of highly fuzzy nucleosomes in the gene bodies that were almost absent in promoter regions (Fig. 1f).

Altogether, these results indicated that the naked DNA correction significantly improved nucleosomal mapping. We wondered whether using gently digested MNase-seq data would introduce relevant alterations into the assigned positions of nucleosomes. To test this, we compared our map to those obtained by the Widom lab by following an in vivo chemical method that did not require the use of MNase [31]. Comparisons were made as described in the Methods section. As a reference, we included the map obtained by the Rando lab from the extensively digested mononucleosomes [12]. As shown in Additional file 3 A, the non-corrected gently digested MNase-seq map exhibits larger differences with the Widom map than with the Rando map. However, the naked DNA correction increased the similarity of our gently digested MNase-seq map to the Widom map, and at the same level as the Rando–Widom comparison (Additional file 3 A, B). So, by subtracting the MNase sequence bias, we generated a nucleosomal map, which was as precise as at least those obtained from extensively MNase-digested samples, and we expected to preserve the MNase-sensitive nucleosomes potentially produced by the dynamic chromatin phenomena, which occur during gene transcription.

### Naked DNA correction standardizes fragile nucleosomes

**Fig. 2:**
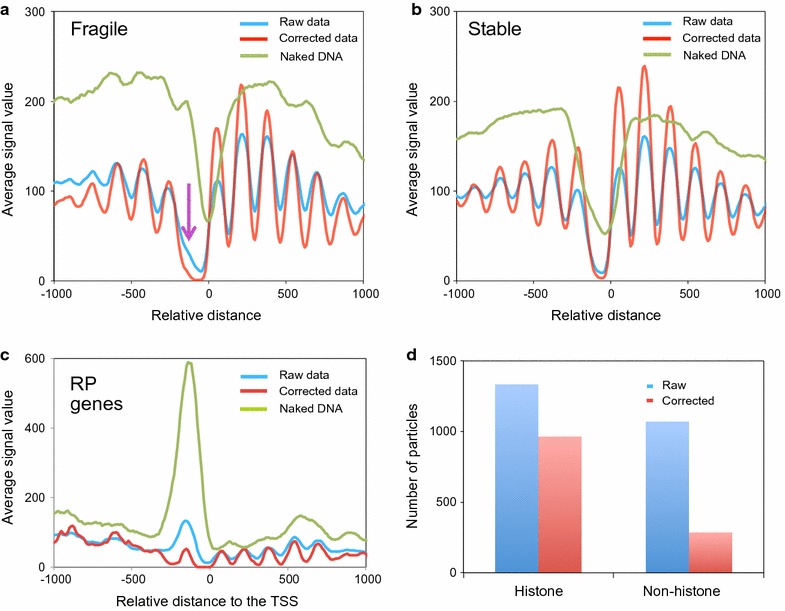
Naked DNA correction promotes standardization of fragile nucleosomes. **a** The metagene analysis of nucleosomal occupancy in 1856 genes that contained a fragile − 1 nucleosome, according to [17]. Genes were scaled to the same length and then aligned to their TSS. The purple arrow indicates the expected position of fragile nucleosomes. **b** The metagene analysis of nucleosomal occupancy in 2872 genes that contained a stable − 1 nucleosome, according to [17]. **c** The metagene analysis of nucleosomal occupancy in RP genes. **d** A comparison of the effect of the naked DNA correction on the detection of histone and non-histone MNase-sensitive particles, according to the data from [43]

MNase-sensitive nucleosomes have been described in a number of gene promoters and terminators across the yeast genome [12, 17, 24]. The detection of such fragile structures has sometimes been elusive and seems to depend strongly on the degree of MNase digestion. Using our raw and corrected data, we generated the nucleosomal profiles of the set of genes that contain fragile nucleosomes immediately upstream of the TSS according to the definition of [17]. In the profile made from the raw data, we detected a small shoulder on the proximal side of the first nucleosome upstream of the NDR (the purple arrow in Fig. 2a), which was not present in the control genes (Fig. 2b). The fact that we did not detect a defined nucleosomal peak at this position is likely due to the gene-to-gene variability observed for the distance from these MNase-sensitive structures to the TSS [24]. When we focused on ribosomal protein (RP) genes (see gene category lists in Additional file 4), the category with the maximal frequency of these fragile nucleosomes, we detected a very prominent nucleosomal peak at this position of the raw metagene (Fig. 2c). However, the magnitude of this peak was softened by the naked DNA correction (Fig. 2c). Similarly, the above-mentioned shoulder of the general raw metagene of the fragile nucleosome-containing genes disappeared in the corrected profile, which resulted in a broader NDR than that observed in the control set of genes (Fig. 2a, b). So, the naked DNA correction had a marked impact on the profile of the fragile nucleosome-containing promoters. This is explained by the very strong resistance exhibited by naked DNA to the MNase upstream of the TSS in this kind of promoters, which generated nucleosome-like DNA fragments that were detected most clearly by DANPOS at this position (Fig. 2a, c), but were not detected in the control genes (Fig. 2b). This resistance is likely due to the presence of DNA sequences that disfavor MNase cutting [37]. To test if sequence composition could explain the biased distributions of the nucleosome-like fragments in the naked DNA data, we plotted the naked DNA signals against their G + C content. We found a significant positive correlation with a higher frequency of MNase-resistant fragments that exhibited G + C contents above the average value of the yeast genome (Additional file 5 A).

Correcting the MNase sequence preference is, therefore, useful for establishing the real occupancy of fragile nucleosomes in comparison with nearby nucleosomes, which are not similarly affected by the MNase sequence bias. This is well exampled by the RP metagene, where the naked DNA correction lightened the weight of the − 1 nucleosome and, at the same time, uncovered a clear array of nucleosomes in the gene body, which enhanced the rather subtle profile of their raw chromatin (Fig. 2c and Additional file 5 B). This patent difference between raw and corrected profiles was also detected when we extended the metagene to all the Rap1-driven genes (Additional file 5 C), but came closer to the average in the genes driven by Abf1 and Reb1 (Additional file 5 D, E). All three transcription factors have been related to fragile nucleosomes [17].

The strong resistance to MNase of some of these promoter regions may even lead to map nucleosome at a site that is not occupied by histones. This has been elegantly shown by [43]. We analyzed the list of histone and non-histone MNase-sensitive structures defined in this work and found that the naked DNA correction eliminated most of the non-histone nucleosome-like particles from our refined map, but maintained the majority of the real MNase-sensitive nucleosomes (Fig. 2d). The differential action of the naked DNA correction on histone and non-histone MNase-sensitive particles was highly significant (Fisher’s exact test *p* < 0.01). In contrast, we found that the naked DNA correction had no particular effect on the profiles of those genes that contained RSC-bound asymmetric nucleosomes at their promoters [44]. This finding indicates that they are not specifically affected by the sequence preference of MNase (Additional file 5 F, G).

### The naked DNA correction is particularly important for canonical TATA genes

**Fig. 3:**
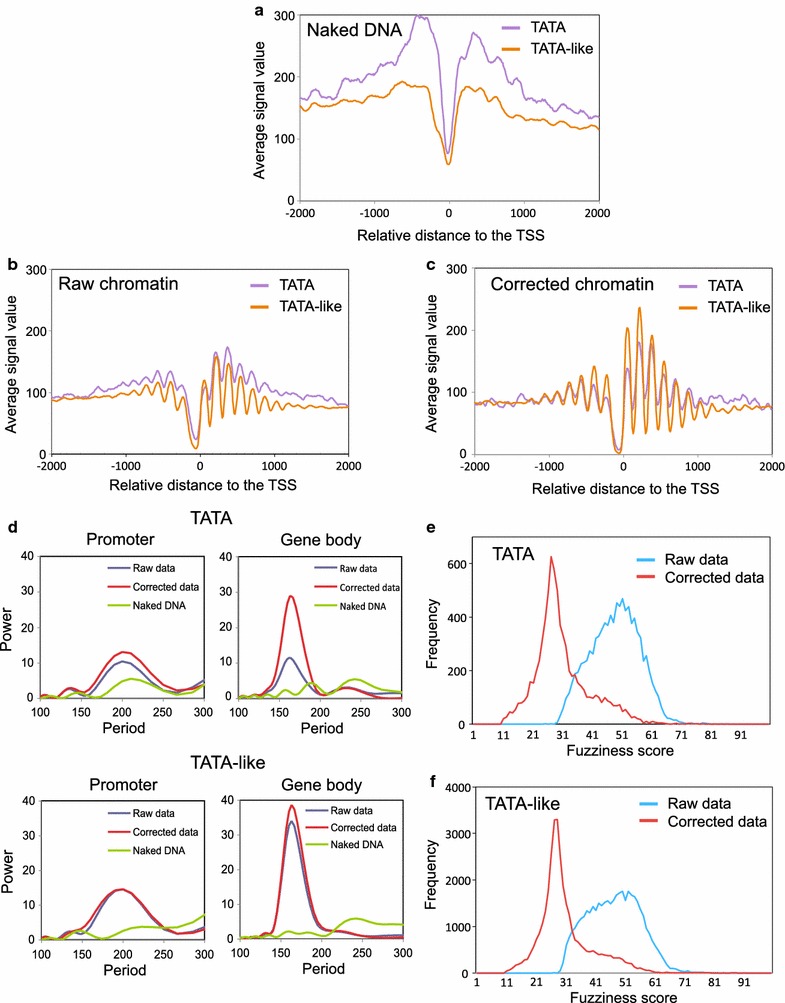
TATA gene bodies are particularly responsive to naked DNA correction. **a**–**c** The metagene analysis of the occupancy of the naked (**a**), raw (**b**), and corrected (**c**) data by comparing the TATA (purple) and TATA-like (orange) genes. After classification, genes were aligned to their TSS, as in Fig. 1a. **d** A spectral analysis of the promoter and gene body nucleosomes of the TATA and TATA-like genes. Details as in Fig. 1c, d. **e**, **f** The fuzziness score distribution before (blue) and after correction (red) in the TATA-containing (**e**) and TATA-like genes (**f**)

We wondered whether, in addition to MNase-sensitive nucleosomes, the sequence bias would affect some particular type of genes more than others. It has been described that the kind of TATA elements present in core promoters influences their nucleosomal configuration [23]. So, we decided to classify genes according to the structure of their core promoters. We found major differences between the MNase patterns of the genes that contained a canonical TATA box and those with a TATA-like element (Fig. 3a–c). The naked DNA of TATA genes displayed more resistance to MNase both upstream and downstream of the TSS compared to the TATA-like genes, inducing the bioinformatic tool to detect more nucleosome-like peaks in that type of genes (Fig. 3a). Consequently, the TATA and TATA-like metagenes obtained from the raw chromatin data also displayed quite different profiles (Fig. 3b). In contrast, the metagenes that resulted from the corrected chromatin data were rather similar, and the differences between the TATA and TATA-like corrected profiles concentrated in the four nucleosomes surrounding the NFR (lesser occupancy of the − 2 to + 2 nucleosomes in the TATA genes) (Fig. 3c). This situation agrees well with the competition between the preinitiation complex and the TSS-proximal nucleosomes described for TATA genes [23].

Interestingly, the differential sensitivity to MNase between the TATA and TATA-like naked DNAs was also evidenced around the pAS (Additional file 6 B). As a result, the naked DNA correction had a stronger impact on the pAS region of the TATA metagene than on the TATA-like one (Additional file 6 A). The spectral analysis of the TATA and TATA-like genes also showed that the difference between raw and corrected data was stronger for the TATA genes than for the TATA-like ones. This was especially visible in the gene body nucleosomes (Fig. 3d). All these differences made the correction of the DNA sequence bias of MNase especially important for the TATA genes.

We wondered whether the stronger impact of the naked DNA correction on TATA genes could be due to a differential distribution of the highly and poorly transcribed genes between TATA and TATA-like genes. We divided all the genes into four quartiles according to their transcription rates [45] and compared the effect of the naked DNA correction on the TATA and TATA-like genes within each quartile. When we focused on the most highly transcribed quartile (Q1), the profile of TATA genes was more clearly improved by the correction than the TATA-like one, particularly in the gene body (Additional file 6 A). The opposite was observed in the least transcribed quartile (Q4), where the TATA gene profile was almost not affected by correction, while the TATA-like one clearly was (Additional file 6 A). In fact, the naked DNA correction equally affected the TATA-like genes in all four quartiles, whereas significant differences were observed in TATA profiles (Additional file 6 A). These results indicate that the differential effect of the naked DNA correction on the nucleosomal profiles of TATA and TATA-like genes is not merely explained by the transcription level, but somehow contributes to discriminate the effect of the naked DNA correction within TATA genes.

Since the MNase bias is sequence dependent, these last results predicted differential sequence features between the TATA and TATA-like genes. A close analysis of the sequences of these two groups of genes revealed that their position-dependent nucleotide composition exhibited some dissimilarity. In addition to the expected differences in the core promoter, the TATA and TATA-like genes displayed differential nucleotide profiles both upstream and downstream of that region (Additional file 7 A). In the upstream promoter region, the coding strands of TATA genes were significantly poorer in A and T than those of the TATA-like ones and were reciprocally richer in G and C (Additional file 7 B). This difference could merely reflect local changes in nucleotide composition due to the presence of regulatory upstream activating sequences. However, we also found significant differences between the coding strands of the TATA and TATA-like in gene bodies, particularly in their A and C contents (Additional file 7 B). These differences could well explain the stronger effect of the naked DNA correction on TATA genes.

To complete the comparison between the TATA and TATA-like genes, we contrasted nucleosomal fuzziness in raw and corrected data. The fuzziness score distributions of the TATA and TATA-like genes in raw data clearly differed in shape (Fig. 3e, f). We calculated an average score of 47.93 for TATA genes, which was significantly higher than the 46.61 average of the TATA-like genes (Student’s *t* test *p* < 0.001). When we compared the corrected data, we found that the two groups of genes showed much more similar profiles and nonsignificantly different average scores: 30.23 and 30.26 for the TATA and the TATA-like genes, respectively (Student’s *t* test *p* > 0.05) (Fig. 3e, f). We conclude that the correction of the MNase sequence bias is essential to accurately map nucleosomes in canonical TATA genes.

### TFIIS contributes to nucleosome positioning in gene bodies

Transcription elongation is one of the main biological processes to affect nucleosome dynamics. Therefore, perturbation of transcription elongation is expected to provoke alterations to nucleosome positioning along the gene body. TFIIS is a nonessential RNA-cleavage factor that favors chromatin transcription (see Introduction). The nucleosomal patterns of most genes were not drastically affected by *dst1∆* (the mutant that lacked the TFIIS-encoding *DST1* gene), although we detected 1753 genes with at least one nucleosome with significantly altered occupancy (either positively or negatively), and some individual genes with extensive changes (Additional file 8).

**Fig. 4:**
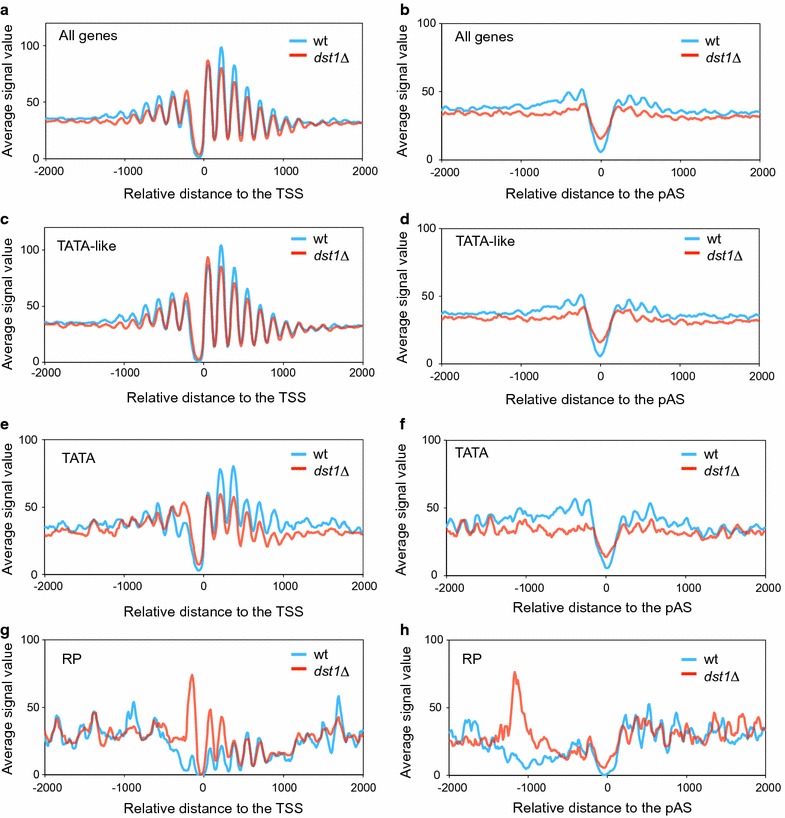
TFIIS influences nucleosomal occupancy across the genome. The nucleosomal metagenes of all the genes (**a**, **b**), TATA-like (**c**, **d**), TATA (**e**, **f**), and RP genes (**g**, **h**) in the wild-type and *dst1∆* cells, aligned to the transcription start site (**a**, **c**, **e**, **g**) or the polyadenylation sequence (**b**, **d**, **f**, **h**) are shown

We wondered whether on top of these particular cases, lack of TFIIS would have a general impact on nucleosome positioning, which could be detected by our MNase-seq refined method. The global metagene profiles showed that this was actually the case. We found that *dst1∆* presented generally reduced nucleosomal occupancy in gene bodies, which was detected when aligned to either the TSS or the pAS (Fig. 4a, b). In the latter, the signal at the 3′ nucleosome-depleted region increased, which indicates defective nucleosome positioning at the end of the transcription units (Fig. 4b).

**Fig. 5:**
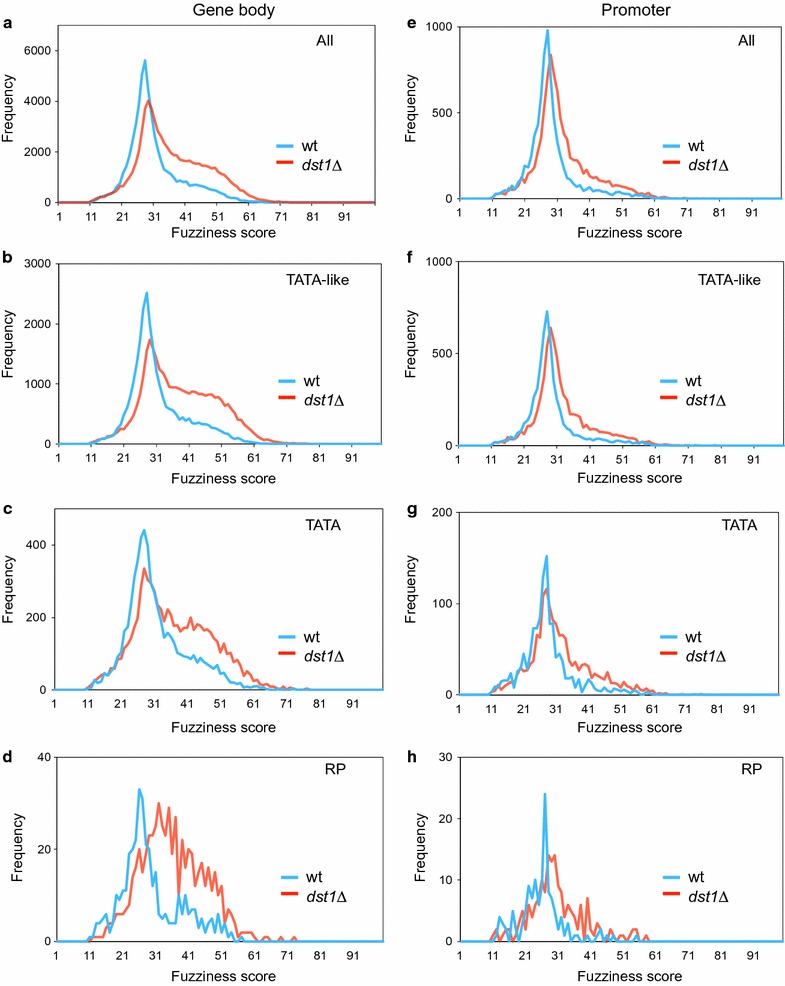
Lack of TFIIS increases nucleosomal fuzziness. Distribution of the fuzziness scores in the wild-type and *dst1∆* cells of all the genes (**a**, **e**), the TATA-like (**b**, **f**), TATA (**c**, **g**), and RP genes (**d**, **h**) in either gene bodies (**a**–**d**) or promoter regions (**e**–**h**)

In spite of being clearly detectable in the TATA-like genes, the impact of *dst1∆* on gene body nucleosomes was particularly strong in TATA genes (Fig. 4c–f). This difference between the TATA and TATA-like genes was clearly visible in both the TSS and pAS metagenes (Fig. 4c–f). *dst1∆* also provoked a net accumulation of highly fuzzy nucleosomes (fuzziness score higher than 40) in the gene bodies (Fig. 5a). The change in the average fuzziness scores of the gene bodies was significant in all the gene categories that we investigated (Student’s *t* test, *p* < 0.001) (Fig. 5a–d), but was stronger in the TATA-like genes (average score of 30.7 in the wt vs. 36.9 in *dst1∆*, Fig. 5b) than in those genes that contained a canonical TATA element (average score of 30.7 in the wt vs. 35.5 in *dst1∆*, Fig. 5c).

These alterations to nucleosome positioning in *dst1∆* agree with the well-known role of TFIIS in transcription elongation. Increased nucleosomal fuzziness in *dst1∆* is likely related to the interference that takes place between nucleosomes and arrested RNA pol II molecules, which need TFIIS to resume elongation by solving its backtracked configuration. Accordingly, the impact of *dst1∆* in promoter regions was less prominent (Fig. 5e) (see below). We plotted the fuzziness scores in relation to the distance to the TSS. WT fuzziness increased from the TSS to around 300 bp in the gene body and then reached a plateau. In contrast, *dst1∆* fuzziness continued to increase all the way to the end. So, after position + 300, the difference in the fuzziness score between *dst1∆* and the wt increased with distance to the TSS (Additional file 9 A). This finding is consistent with the higher incidence of RNA pol II backtracking detected after nucleosome + 2 [5]. A large proportion of these new highly fuzzy nucleosomes (scoring over 40) that *dst1∆* produced in the gene bodies resulted from the alteration of a subset of nucleosomes that obtained low fuzziness scores (below 30) in the wt (Additional file 9 B). This suggests that backtracked RNA pol II molecules can keep nucleosomes significantly shifted from their standard position in the gene bodies.

The above-described results indicate a positive effect of TFIIS on nucleosome positioning in the gene bodies. However, we noted a different behavior for the RP genes in *dst1∆*. In this case, the metagene showed a clear increase in the occupancy of TSS nucleosomes + 1 and + 2 (Fig. 4g) and very mild effects on pAS nucleosomes − 1 and − 2 (Fig. 4h). The average fuzziness score of the RP nucleosomes also significantly increased from 28.1 in the wt to 34.4 in *dst1∆* (Student’s *t* test, *p* < 0.001). In this case, however, the main change in distribution consisted in the accumulation of moderately fuzzy nucleosomes, with scores between 30 and 40 (Fig. 5d).

This different effect of *dst1∆* on RP chromatin would not be due to lower levels of RNA pol II sitting on these genes because we previously showed that they did not significantly lower in this mutant [46] (see also Additional file 10 C). Since TFIIS is a transcription elongation factor, and the RP genes are the most highly transcribed across the genome, we reasoned that their particular fuzziness profile in *dst1∆* could be shared by other highly transcribed genes. We tested this by comparing the fuzziness score distribution of the gene body nucleosomes of the RP genes to the 335 most transcribed non-RP genes [5]. We found that the fuzziness distribution of the highly transcribed gene bodies in *dst1∆* exhibited a closer profile (Additional file 9 C) to the *peak*-*plus*-*shoulder* shape of the whole genome (Fig. 5a) than to the almost symmetric profile of the RP gene bodies (Fig. 5d).

Lack of TFIIS also caused some chromatin alterations in promoter regions. We detected that the occupancy of nucleosome − 1 in the RP metagene significantly increased (Fig. 4g) and was also detected around − 1000 in the pAS alignment (Fig. 4h). This effect of *dst1∆* on nucleosome − 1 was also present in TATA genes (Fig. 4e). In this case, the alteration seemed highly specific of nucleosome − 1 since nucleosomes + 1 and − 2 did not change compared to the wt.

In contrast to occupancy, the nucleosomal fuzziness of promoters did not quantitatively alter more in the RP and TATA genes than in the TATA-like ones accordingly to the fuzziness scores distributions (Fig. 5f–h). The change produced by *dst1∆* was much weaker in promoter regions than in the gene bodies, both globally and in all the tested gene categories (compare Fig. 5a–d to e–h, and Additional file 9 C, D), which suggests that the category-specific nucleosomal changes detected in the gene bodies might reflect differences in chromatin dynamics during transcription elongation (see later).

**Fig. 6:**
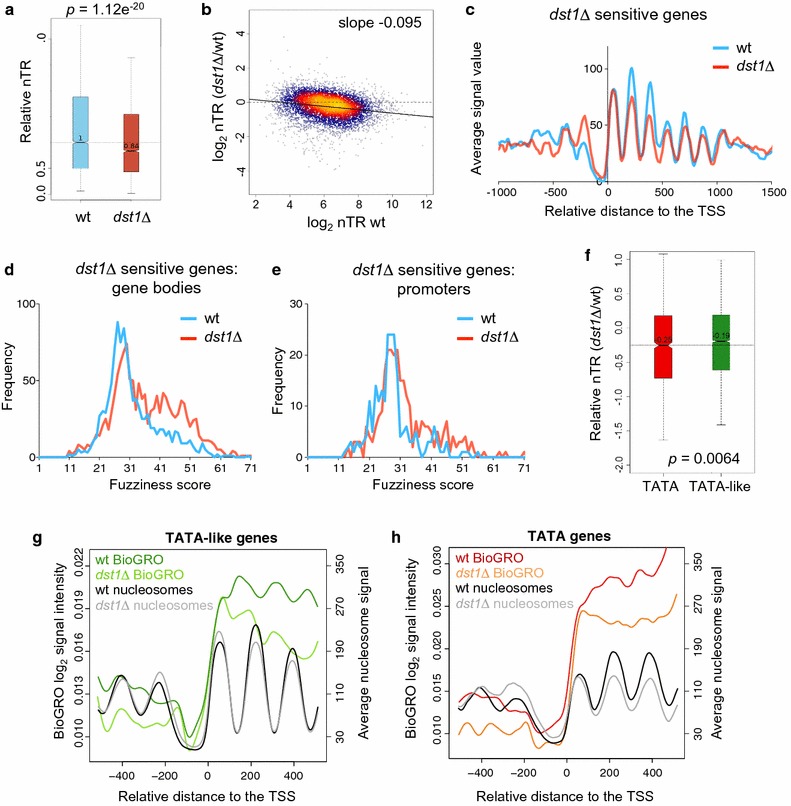
Genes most transcriptionally affected by *dst1∆* exhibit both occupancy and fuzziness nucleosomal alterations. **a** Comparison between the nTR of the wild-type and *dst1∆* cells across the genome. The boxplot compares the median of the two nTR distributions shown in Additional file 10 A. The two distributions significantly differ according to the Wilcoxon–Mann–Whitney U-test. **b** The scatter plot of the *dst1∆*/wt nTR ratio *versus* the wt nTR. **c** Average nucleosomal profile in the wild-type and *dst1∆* cells of those genes whose GRO signal went below one half in *dst1∆*, aligned to the TSS. **d**, **e** Distribution of the gene body (**d**) and promoter (**e**) nucleosomal fuzziness scores of those genes whose GRO signals went below one half in *dst1∆*. **f** Comparison between the *dst1∆*/wt nTR ratios of the TATA and TATA-like genes. The two distributions significantly differ according to the Wilcoxon–Mann–Whitney U-test. **g**, **h** Bio-GRO signals of TATA-like (**g**) and TATA genes (**h**) in the wild type and *dst1∆*. After classification, genes were aligned to their TSS, as in Fig. 1a. The corresponding nucleosomal metagenes are shown as a reference

We previously described a specific link between TFIIS and RP gene transcription, which was specifically enhanced under transcriptional stress conditions [46]. We wondered whether the nucleosomal alterations to TATA genes would also reflect preferential dependence on TFIIS. We analyzed the nascent transcription rates (nTR) of the wt and *dst1∆* cells by genomic run-on (GRO). GRO signals directly reflect active transcription, save RNA polymerases which become inactive by backtracking and remain sitting on the gene body [47]. nTR signals significantly decreased in the *dst1∆* cells (Additional file 10 A) and had a significantly lower median (Wilcoxon–Mann–Whitney U-test, *p* = 1.12 × e^−20^, Fig. 6a). This reduction in nTR provoked by *dst1∆* was particularly strong in the most highly transcribed genes (Fig. 6b). Indeed, the nTR in *dst1∆* decreased in 186 genes by below one-third (− 1.5 log_2_ units) of their wt level. We analyzed the nucleosomal organization of this subset of genes and found two main differences in its TSS metagene: a generally reduced occupancy of gene body nucleosomes (except nucleosome + 1) and greater occupancy of the − 1 nucleosome (Fig. 6c). We also found highly fuzzy nucleosomes (fuzziness scores above 40) in gene bodies (Fig. 6d) and a much less marked increase in the fuzziness of promoter nucleosomes (Fig. 6e). All these nucleosomal alterations resembled those exhibited by TATA genes (Figs. 4e, 5c, g). In fact, this subset of *dst1∆*-sensitive genes was significantly enriched in TATA genes (hypergeometric test, *p* = 0.006) (Additional file 10 B).

We wondered whether these results could reflect a general difference in the dependence on TFIIS between the TATA and TATA-like genes. Although the nTR of both categories was affected by *dst1∆*, the GRO data revealed a significantly stronger effect on the TATA genes (Wilcoxon–Mann–Whitney U-test, *p* = 6.4 × 10^−3^) (Fig. 6f). We also ran a high-resolution analysis of nascent transcription in *dst1∆* following the Bio-GRO protocol described by our laboratories [5]. We found a characteristic anti-nucleosomal pattern in the bio-GRO signal of the TATA-like genes in the wt (Fig. 6g, dark green). This signal slightly lowered in *dst1∆,* but retained the characteristic wavy pattern (Fig. 6g, light green). The signal of the TATA genes in the wt did not exhibit a clear anti-nucleosomal profile, particularly in the nucleosome + 2 region (Fig. 6h, red) and, unlike the TATA-like genes, no clear wavy pattern was observed in *dst1∆* (Fig. 6h, orange). This loss of anti-nucleosomal pattern did not happen in the RP genes (Additional file 10 C). So, although the Bio-GRO signals of all the gene types lowered in *dst1∆*, their profiles indicated qualitative differences in the contribution of TFIIS to transcribe TATA and TATA-like chromatin.

### TATA and TATA-like gene bodies undergo differential chromatin dynamics during transcription elongation

**Fig. 7:**
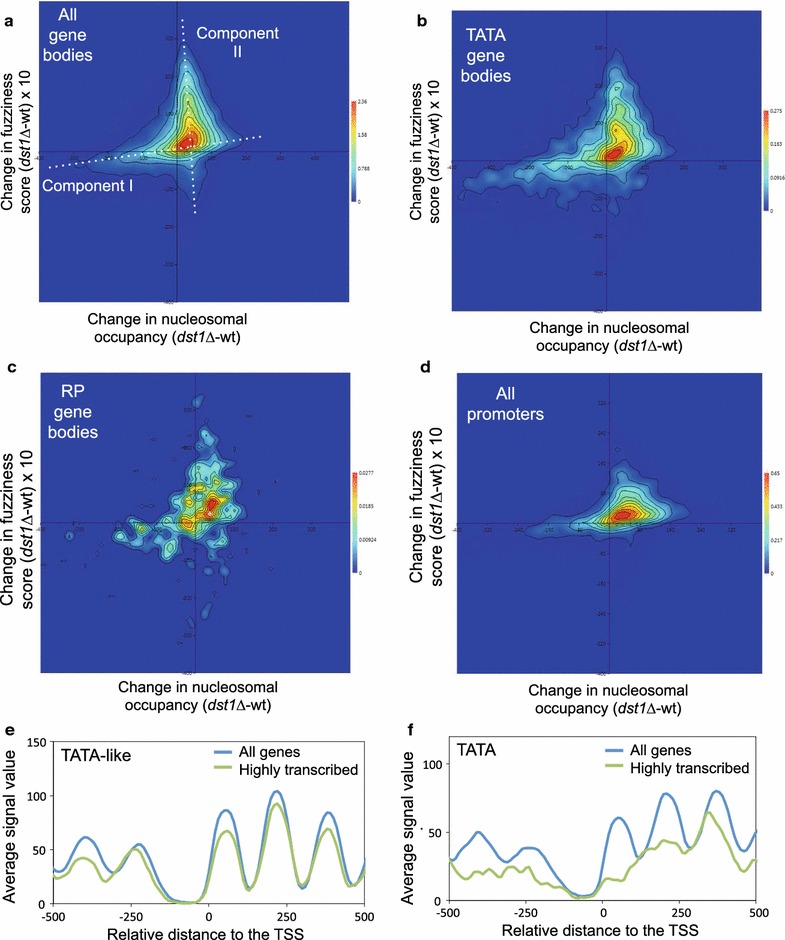
Nucleosomes dominated by increased fuzziness in *dst1∆* segregate from those dominated by decreased occupancy. **a**–**d** Heat maps of the difference in fuzziness *versus* occupancy between the mutant *dst1∆* and the wild type for the gene body nucleosomes of all the genes (**a**), the canonical TATA (**b**) and RP genes (**c**), and for the promoter nucleosomes of all the genes (**d**). **e**, **f** Metagene analysis of the occupancy in all the genes and the 335 most transcribed genes according to the data in [5], divided into the TATA-like (**e**) and TATA-containing (**f**) genes. Promoter nucleosomes were defined as those between the TSS and 500 bp upstream. Gene body nucleosomes were those between the TSS and the STOP codon

To further investigate this potentially dual contribution of TFIIS to chromatin dynamics, we checked whether the changes in fuzziness and occupancy produced by *dst1∆* in gene body nucleosomes were coupled, or simply happened independently. To do so, we plotted the difference between the wt and the *dst1∆* mutant in nucleosome occupancy (peak height) and difference in the fuzziness scores for each gene body nucleosome that we were able to map. We plotted 31,599 nucleosomes in all and found two main components (Fig. 7a). First, we detected a low, but significant, direct correlation between the changes in occupancy and fuzziness (Pearson’s *r* = 0.31, *p* < 0.001) (Fig. 7a). The group of nucleosomes dominated by this component, i.e., those whose occupancy was destabilized by lack of TFIIS, tended to become moderately less fuzzy (Fig. 7a). We also detected a second component, dominated by the nucleosomes that became highly fuzzy in *dst1∆*, that displayed constant and slightly positive change in occupancy (Fig. 7a). The presence of these two components indicates that, for the majority of nucleosomes, lack of TFIIS predominantly changes either occupancy or fuzziness, but not both in parallel and at the same intensity.

When we represented the subset of the nucleosomes mapping on the body of TATA genes, we found that the higher proportion of nucleosomes with lesser occupancy, which characterized this category (Fig. 4e, f), strengthened the first above-described component (Fig. 7b), which led to a slightly higher correlation between the changes in occupancy and fuzziness (Pearson’s *r* = 0.37). In contrast, the nucleosomes on the body of the TATA-like genes showed a lower correlation (Pearson’s *r* = 0.30) and a higher balance between the two components than TATA genes (Additional file 11 A). This difference was not due to the slightly higher average transcriptional activity of TATA genes (17) since the RP gene bodies (the most transcribed subset of the TATA-like genes) exhibited a lower correlation (Pearson’s *r* = 0.32) and were less enriched in the nucleosomes of the first component than TATA genes (Fig. 7c).

Next, we analyzed the effect produced by *dst1∆* on promoter nucleosome occupancy *versus* the difference in the fuzziness scores for each promoter nucleosome (Fig. 7d). The plot reflected less relevance of component II (the nucleosomes that markedly increased fuzziness without changing occupancy) in the promoters than in the gene bodies, which agrees with the low frequency of the highly fuzzy nucleosomes that accumulated in promoter regions in *dst1∆* (Fig. 5e). Component II was also weak in nucleosome + 1 (Additional file 11 B), which indicates that it is a consequence of productive elongation rather than of promoter escape.

These differences between the TATA and TATA-like genes in *dst1∆* suggest the existence of two alternative ways of chromatin dynamics during transcription elongation: one connected with nucleosome eviction, which would also operate in promoter regions, and another one that would involve nucleosome remodeling without eviction. If such an alternative transcriptional dynamics exists, heavy transcription should increase the difference between the metagene profiles of the TATA and TATA-like genes. Figure 6e, f shows that this was actually the case. The most highly transcribed (the highest decile) TATA-like metagene retained the periodic wavy profile of all the TATA-like genes (Fig. 7e), while the most highly transcribed TATA metagene presented a much noisier profile than the whole set of the TATA genes both upstream and downstream of the TSS (Fig. 7f).

## Discussion

The changes in nucleosome positioning associated with the functional transactions of the genome can be very subtle [48]. In order to detect and define such changes, considerable efforts are being made to improve the accuracy of nucleosome-mapping methods. Resolution has certainly improved thanks to deep sequencing techniques. However, as long as MNase is the main nucleosome-mapping tool, its known sequence bias remains an issue. The most widespread way used to date to minimize sequence bias is overdigestion [21], which has been demonstrated to impair the detection of some nucleosomes, particularly in promoter regions [12, 17]. The easy method that we developed, which employs partially digested material and a correction based on a naked DNA analysis, is an alternative to overdigestion and accounts for the sequence bias of MNase. Moreover, as the cells used to extract naked DNA were treated by the same procedure as with chromatin samples (fixation, protoplast generation, permeabilization), any other nucleosome-unrelated cut that DNA could undergo during this process would also be filtered out. Naked DNA digestion has been previously utilized to control MNase experiments [36]. Here, we used the information obtained from digested naked DNA to refine nucleosomal mapping. This information was collected from a single level of naked DNA digestion. We are aware that this method could be further refined by performing the kinetics of naked DNA digestion and comparing correction outputs. Other aspects of the method can also be improved, like the stringency of the peak definition of DANPOS, which seems to be the reason for some real nucleosomes being misdetected, according to the relatively small number of mapped nucleosomes (45,000). Yet, despite these defects, we consider that this first approach was sufficient to demonstrate that the mild digestion and naked DNA correction combination is useful for generating high-quality nucleosomal occupancy maps, improves the analysis of the MNase-sensitive structures, and provides an optimal quantification of nucleosomal fuzziness. The simplicity of this method makes it easily applicable to any organism and can prove useful in other procedures where light MNase digestion is needed, like whole-epigenome profiling [49].

This improvement proved particularly significant in the genes that contained a canonical TATA element. We detected some differences in the nucleotide composition of the TATA and TATA-like genes, in both promoter regions and gene bodies (Additional file 7), which could explain this differential action of MNase (Fig. 3). The contribution of the gene sequence to nucleosome positioning is not mediated directly by nucleotide composition, but by periodic occurrences of dinucleotides [50, 51]. Therefore, two sequences may similarly favor the positioning of nucleosomes, but their sensitivity to MNase might differ.

This improved method is sensitive enough to detect subtle global changes in nucleosome positioning, like those caused by lack of TFIIS (Figs. 4, 5). We observed an overall decrease in nucleosomal occupancy in parallel to increased nucleosomal fuzziness. The accurate quantitation of nucleosomal fuzziness that the improved method enables was essential to detect this general influence of TFIIS on nucleosome positioning.

We detected alterations to both gene bodies and promoter regions. These alterations were remarkably similar to those produced by lack of Isw1 and Chd1, two factors that contribute to maintain nucleosome spacing across the genome [52]. Interestingly, *isw1∆* and *dst1∆* exhibit a positive genetic interaction [53], which suggests that the restrictions to nucleosome mobility caused by *dst1∆* in the transcribed gene bodies might be compensated by the relaxed spacing of *isw1∆*.

Changes produced by *dst1∆* were particularly clear in the occupancy of the − 1 nucleosomes of TATA and RP promoters (Fig. 4e, f), associated with a minor increase in nucleosomal fuzziness (Fig. 5g, h). This influence of TFIIS on promoter chromatin could be related with the functional interaction of TFIIS to the mediator during preinitiation complex assembly [54–56]. This interaction has been described as being important for PIC assembly, transcription initiation, and for transcription through nucleosome + 1 [57].

More prominent were the alterations observed in the gene bodies, on the nucleosomal map and in the Bio-GRO study. They are consistent with the TFIIS function during transcription elongation and match the results of the in vitro experiments that demonstrated a functional role of TFIIS in the transcription of nucleosomal DNA templates [10, 58, 59]. Our results extend this conclusion to the in vivo situation and uncover a positive contribution of TFIIS to nucleosome positioning. However, in *dst1∆* we did not detect the chromatin relaxation that has been observed when transcriptional activity is absent [12, 17], which suggests that TFIIS does not participate in the retrograde packaging that RNA pol II-dependent transcription exerts on gene body nucleosomes.

**Fig. 8:**
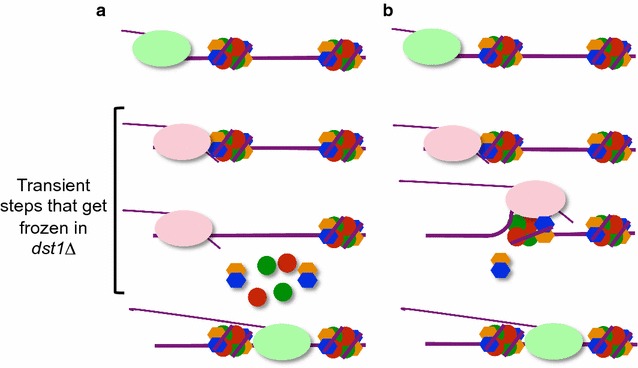
Two alternative modes of chromatin transcription. Our results support the existence of two different modes of chromatin transcription: **a** full histone eviction, followed by reassembly; **b** nucleosomal survival, likely associated with the transient eviction of H2A/H2B dimers. Lack of TFIIS contributes to extend the duration of transient steps in both modes of chromatin transcription

Lack of TFIIS has been demonstrated to prolong the duration of RNA backtracking events [60] and to freeze the transient RNA pol II-nucleosome interactions that take place during transcription elongation, which would otherwise be masked by their fast resolution [31]. The chromatin landscape of *dst1∆* therefore helps to visualize the transient chromatin states that result from nucleosomal dynamics during transcription elongation. We detected some changes in the occupancy of the gene body nucleosomes in *dst1∆*, as well as a very general increase in their nucleosomal fuzziness. Our interpretation is that these two types of changes, respectively, reflect the two main consequences of transcription through chromatin: nucleosome eviction, which necessarily causes reduced occupancy; and nucleosomal sliding, which should lead to higher fuzziness scores. Our analysis showed a poor correlation between changes in occupancy and fuzziness: the fuzziness scores of those nucleosomes with significantly lower levels of occupancy in *dst1∆* slightly diminished, whereas the occupancy of most nucleosomes with a clear increase in fuzziness did not rise proportionally (components I and II of the plot shown in Fig. 7a). This suggests two alternative modes of chromatin dynamics during transcription elongation. Component I would reflect a mode characterized by full nucleosome disassembly in front of the elongating RNA pol II molecule, and subsequent nucleosome reassembly to the back of it (Fig. 8a). In contrast, component II would reflect an alternative mode, associated with nucleosomal sliding, which would involve the transcription of a remodeled nucleosome without full histone eviction (nucleosomal survival) (Fig. 8b). The predominant association of component II with gene bodies rather than with promoter regions (Fig. 7a–d) fits in well with this interpretation. High transcription elongation rates have been usually associated with intense nucleosome eviction in vivo [61]. However, chromatin transcription without full histone eviction has also been described and characterized in vitro [59, 62–64] and has been mechanistically linked to RNA pol II backtracking during transcription elongation [65]. Our results support the notion that nucleosomal survival is also a significant mode of chromatin dynamics linked to transcription elongation in vivo.

The strongest drop in nucleosomal occupancy produced by lack of TFIIS was detected in the body of the TATA genes (Fig. 3e, f). The functional significance of this result was confirmed by analyzing the genes most affected by lack of TFIIS in GRO assays, which were significantly enriched in TATA genes (Additional file 9 B). The occupancy-diminished nucleosomes of the TATA gene bodies did not obtain higher fuzziness scores in *dst1∆* (Fig. 7b), which suggests that classical nucleosome eviction operates in TATA genes during transcription elongation. This agrees with the poorly positioned profile exhibited by the highly transcribed TATA metagene compared with the whole set of TATA genes (Fig. 7e). In contrast, the heavily transcribed TATA-like genes retained the strong positioning of the whole set of TATA-like genes (Fig. 7f). Moreover, the bodies of the RP genes (the most transcribed subset of the TATA-like genes) had moderately higher nucleosome occupancy levels in *dst1∆* (Fig. 4g, h), which were associated with increased fuzziness scores (Fig. 5d), and suggest greater predominance of nucleosomal survival in RP transcription. This divergence between the RP and TATA genes agreed with the differential consequences that lack of TFIIS provoked in these two groups of genes under transcriptional stress [46]. The different effect of *dst1∆* in the Bio-GRO profiles of the TATA and TATA-like genes supports these distinct chromatin dynamics (Fig. 6g, h). Nevertheless, we must underline that the differences between the TATA and TATA-like genes were not extreme, which indicates that these two putative dynamics would co-exist across the genome.

## Conclusions

The combination of partial MNase digestion and naked DNA correction of the sequence bias generates a precise nucleosomal mapping method that optimizes the detection of those altered nucleosomes that are sensitive to MNase and to distinguish them from non-histone MNase-resistant structures. In *Saccharomyces cerevisiae,* canonical TATA genes are particularly sensitive to the MNase sequence bias, which is likely due to their different nucleotide composition. The simplicity of this method makes it easily adaptable to any other organism, independently of the nucleotide composition of its genome.

This method proved useful to detect the subtle alterations in nucleosome positioning produced by lack of transcription elongation factor TFIIS. Differences in both nucleosome occupancy and fuzziness were detected across the genome of the *dst1∆* strain compared to the wild type. The analysis of these alterations uncovers a general contribution of TFIIS to the chromatin landscape and confirms the importance of this factor for gene transcription in vivo.

Lack of TFIIS also facilitates the detection of the transient chromatin configurations that characterize transcription elongation due to the increased duration of backtracking events. Two main types of altered nucleosomes are generated during transcription elongation, dominated by either decreased occupancy or increased fuzziness. The first kind of alteration is more frequent in canonical TATA genes, which suggests the more marked relevance of nucleosome eviction in this gene category. In contrast, fuzzy nucleosomes, which remain in place, characterize the transcription of the TATA-like genes, including the most highly expressed RP genes. This evidence supports the importance of nucleosome survival during chromatin transcription for a very relevant fraction of the yeast genome.

## Additional files

**Additional file 1.** Overview of the method. A) A diagram with the main protocol steps is shown. The fragments to be sequenced were isolated from an ethidium bromide-stained gel (see the example in the figure). The naked DNA samples were visually matched to the chromatin samples by choosing those with a similar maximum fragment size (arrow). Then, the mononucleosome-sized fragments (squares) were isolated. B) The chromatin (blue and red) and naked DNA signals (green) over the *STL1* gene are shown as examples of the results, analyzed by qPCR. The chromatin data are presented before (blue) and after (red) the naked DNA correction. C) The naked DNA signal in the *STL1* gene from different *Saccharomyces cerevisiae* strains. The qPCR results from each naked DNA digestion were standardized and represented as z-scores. D) Some examples of the results analyzed by massively parallel sequencing. The profiles represent the density of nucleosomes dyad axes calculated by DANPOS.

**Additional file 2.** Metagene analysis of the chromatin and naked DNA signals. A, B) Genes were scaled to the same length and then aligned to their TSS or their pAS. All the genes in the yeast genome for which a TSS was available were considered. Zoom-in view of the data in Fig. 1a: A) closer to the TSS; B) closer to the pAS. C) Those genes whose pAS was at least 500 bp away from a TSS were selected, scaled to the same length, and represented as in B. D) Difference between the corrected and raw signals. Genes were scaled and aligned as in Fig. 1a, b. The Y-axis represents the logarithm of the p value of the difference. Two different curves are shown: one represents the positive difference values, i.e., those in which the raw signal was higher than the corrected signal (Dif +), and the other represents the negative difference values, i.e., those in which the raw signal was smaller than the corrected signal (Dif −).

**Additional file 3.** Comparison with chemical mapping method. A) Center-to-center distance of the nearest nucleosome in: the raw data presented here against a chemical modification-based map [31] (blue line), the corrected data against the same reference map [31] (orange line), or the chemical modification-based map against a map that was generated by extensive digestion with MNase [12]. B) Cladogram showing the distance between the different maps mentioned in A.

**Additional file 4.** Genes included in the different categories analyzed in this work.

**Additional file 5.** A metagene analysis to compare the sequencing data before and after correction in different groups of genes. A) A 2D plot to compare the log_10_ signal intensity in the naked DNA sample and the GC content of fragments (normalized by subtracting the genomic average). Pearson’s correlation is shown (*p* < 0.001). B) The metagene analysis of the region around the TSS of the ribosomal protein genes (blue before the correction, red afterward). RP genes were scaled to the same length and then aligned to their TSS. C, D, E) The metagene analysis of the 1363 genes bound by Rap1 (C), the 1311 genes bound by Abf1 (D) and the 281 genes bound by Reb1 (E), according to the DNA binding data from http://www.yeastract.com/. F, G). The metagene analysis of 115 and 392 genes that, respectively, contained a − 1 (F) or + 1 asymmetric nucleosome (G), according to the data from [44].

**Additional file 6.** A metagene analysis to compare the sequencing data before and after the correction in TATA genes versus TATA-like genes A) The metagene analysis of the chromatin (blue before the correction, red afterward) and the naked DNA signals (green) around the pAS in the TATA (left panel) and TATA-like genes (right panel). Genes were scaled to the same length and then aligned to their pAS. B) Genes were divided into quartiles according to their transcription rate [45] and then further subdivided into TATA or TATA-like genes. All the resulting eight groups were scaled and aligned to their TSS. The chromatin signal before and after correction is shown.

**Additional file 7.** Nucleotide composition of the sequence of the TATA and TATA-like genes. A) Frequency of each nucleotide in the TATA (red) and TATA-like genes (blue) at each position in relation to the TSS. B) The average nucleotide frequency in the promoter (− 500 to − 100) and the gene body (50–500) of the TATA and TATA-like genes. A Student’s *t* test was applied to compare the TATA and TATA-like genes. S indicates that the difference is significant (*p* < 0.001). *N* indicates that the difference is not significant (*p* > 0.001).

**Additional file 8.** Nucleosome positioning of genes with significant changes between wt and dst1∆. Genes were ordered by the number of nucleosomes that changed (in occupancy or fuzziness) between the wt and *dst1∆*. The nucleosomal profile of the top five genes is presented.

**Additional file 9.** Nucleosome fuzziness in the wt and dst1∆. A) The metagene analysis of the fuzziness score of the wt (blue) and *dst1∆* (red) nucleosomes around the TSS. Genes were scaled to the same length and then aligned to their TSS. B) The change in fuzziness score between the wt and *dst1∆*. Heat map of the fuzziness score of the gene body nucleosomes in the wt and *dst1∆* mutant. Color represents density, which increases from blue to red. The red square highlights those nucleosomes below 40 in the wt and above > 40 in the mutant. C) The fuzziness score distribution of the nucleosomes in the gene bodies of the highly transcribed genes of the wt (blue) and *dst1∆* (red). D) The fuzziness score distribution of the nucleosomes in the promoters of the highly transcribed genes of the wt (blue) and *dst1∆* (red).

**Additional file 10.** Effect of the absence of TFIIS on the expression of the different types of genes. A) Scatter plot of the nascent transcription rate of each gene in the wt (X-axis) and in *dst1∆* (Y-axis). B) Diagram showing the relationship between the group of genes with a weaker GRO signal in *dst1∆* compared to the wt (TFIIS-dependent) in the TATA-containing genes and TATA-like genes. The TATA-containing genes are overrepresented in the TFIIS-dependent genes (hypergeometric test, *p* = 0.006), while the TATA-like genes are under-represented (hypergeometric test, *p* = 0.028). C) The Bio-GRO signals of the RP genes in the wt and *dst1∆*. After classification, genes were aligned to their TSS.

**Additional file 11.** Occupancy-versus-fuzziness changes of the TATA-like gene bodies and + 1 nucleosomes. Heat maps of the difference between the mutant *dst1∆* and the wt in fuzziness *versus* occupancy for the gene body nucleosomes of the TATA-like genes (A), and for the + 1 nucleosome (defined as that between the TSS and 200 bp downstream) of each gene (B).

## Abbreviations

GRO: genomic run-on
MNase: micrococcal nuclease
NDR: nucleosome-depleted region
nTR: nascent transcription rate
pAS: polyadenylation site
RP: ribosomal protein
TSS: transcription start site
wt: wild type
