# Supplementary figures and images for: Subtracting the sequence bias from partially digested MNase-seq data reveals a general contribution of TFIIS to nucleosome positioning
Source: Epigenetics Chromatin. 2017 Dec 7;10:58. doi: 10.1186/s13072-017-0165-x (PMC5719526; doi:10.1186/s13072-017-0165-x)

## Additional file 1

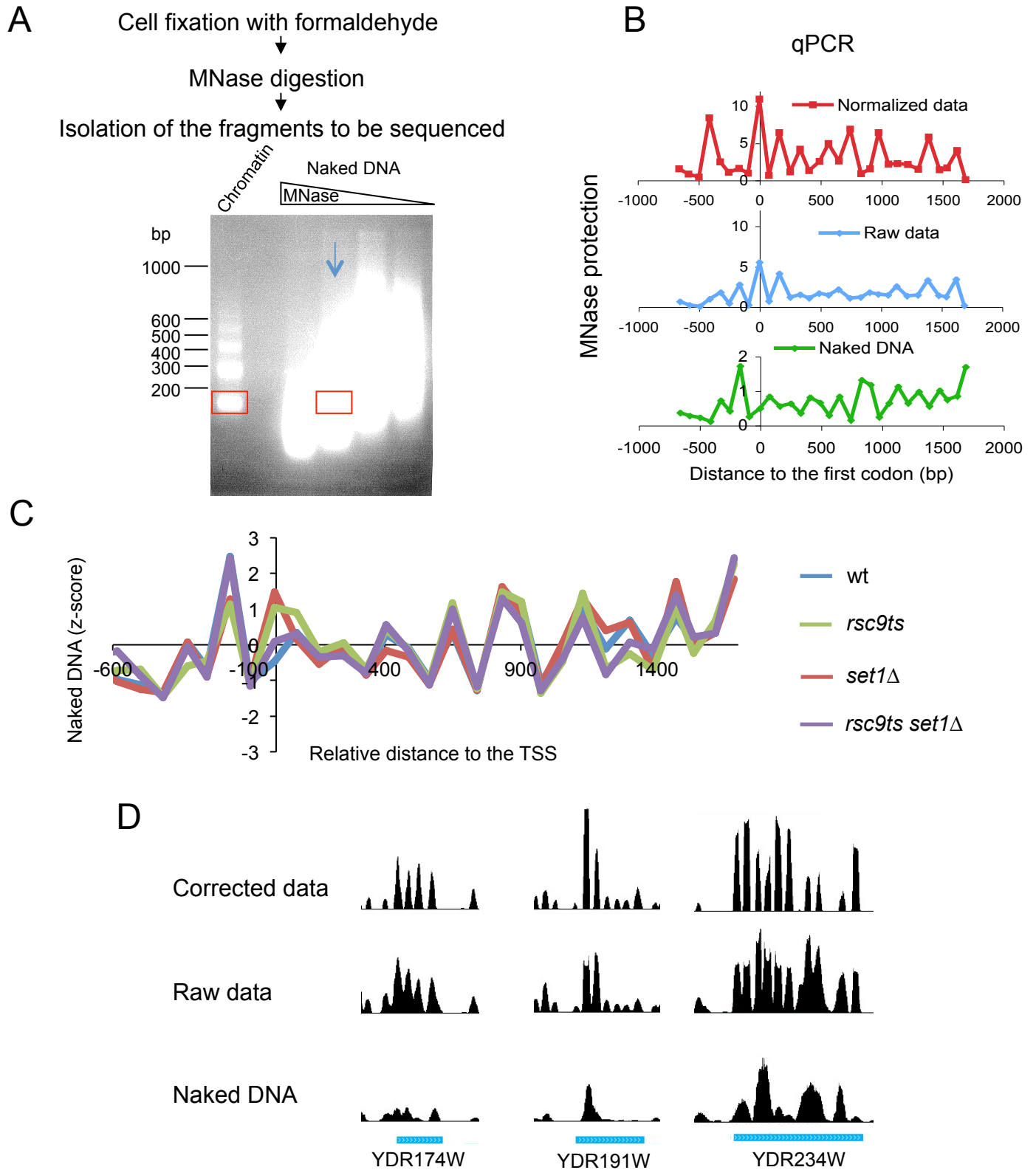

Supplement: Supplementary file 1 — Additional file 1. Overview of the method. A) A diagram with the main protocol steps is shown. The fragments to be sequenced were isolated from an ethidium bromide-stained gel (see the example in the figure). The naked DNA samples were visually matched to the chromatin samples by choosing those with a similar maximum fragment size (arrow). Then, the mononucleosome-sized fragments (squares) were isolated. B) The chromatin (blue and red) and naked DNA signals (green) over the STL1 gene are shown as examples of the results, analyzed by qPCR. The chromatin data are presented before (blue) and after (red) the naked DNA correction. C) The naked DNA signal in the STL1 gene from different Saccharomyces cerevisiae strains. The qPCR results from each naked DNA digestion were standardized and represented as z-scores. D) Some examples of the results analyzed by massively parallel sequencing. The profiles represent the density of nucleosomes dyad axes calculated by DANPOS. [file 13072_2017_165_MOESM1_ESM.pdf]

## Additional file 2

A

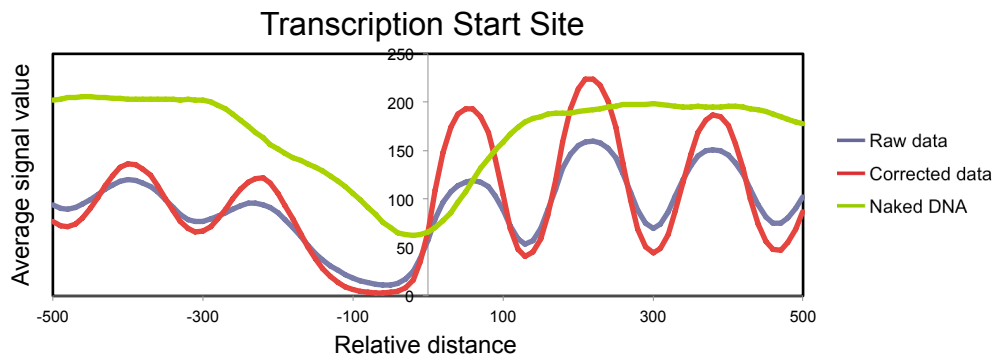

B

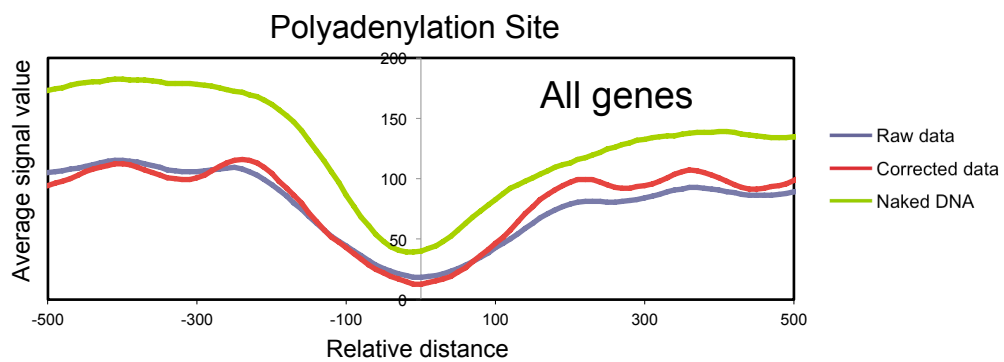

C

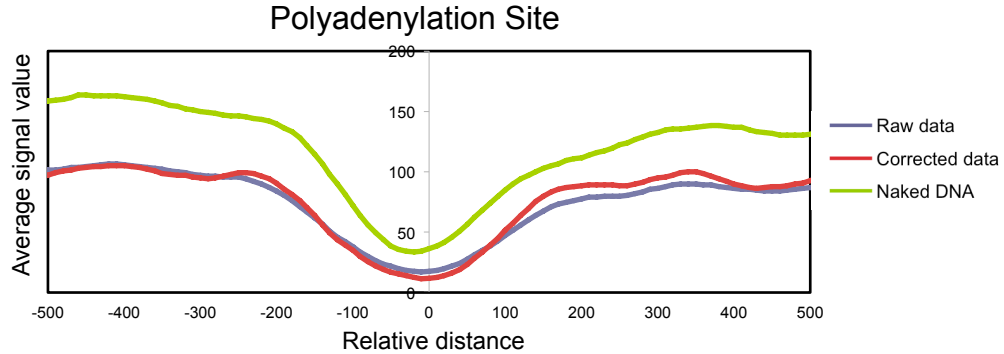

D

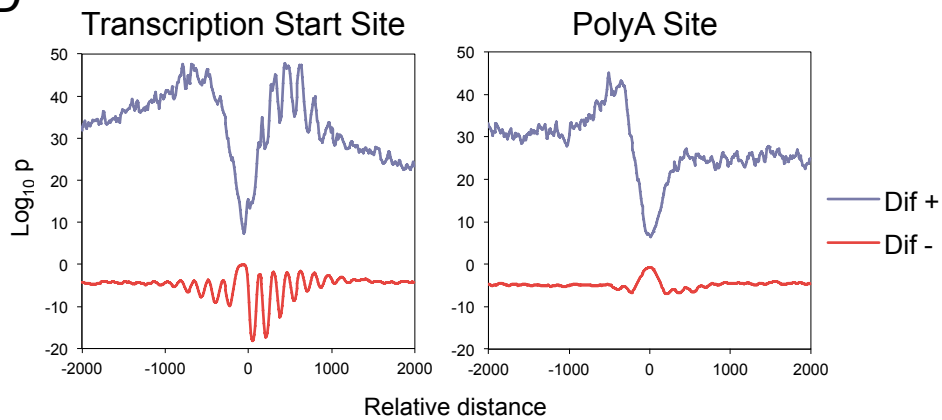

Supplement: Supplementary file 2 — Additional file 2. Metagene analysis of the chromatin and naked DNA signals. A, B) Genes were scaled to the same length and then aligned to their TSS or their pAS. All the genes in the yeast genome for which a TSS was available were considered. Zoom-in view of the data in Fig. 1a: A) closer to the TSS; B) closer to the pAS. C) Those genes whose pAS was at least 500 bp away from a TSS were selected, scaled to the same length, and represented as in B. D) Difference between the corrected and raw signals. Genes were scaled and aligned as in Fig. 1a, b. The Y-axis represents the logarithm of the p value of the difference. Two different curves are shown: one represents the positive difference values, i.e., those in which the raw signal was higher than the corrected signal (Dif +), and the other represents the negative difference values, i.e., those in which the raw signal was smaller than the corrected signal (Dif −). [file 13072_2017_165_MOESM2_ESM.pdf]

# Additional file 3

A

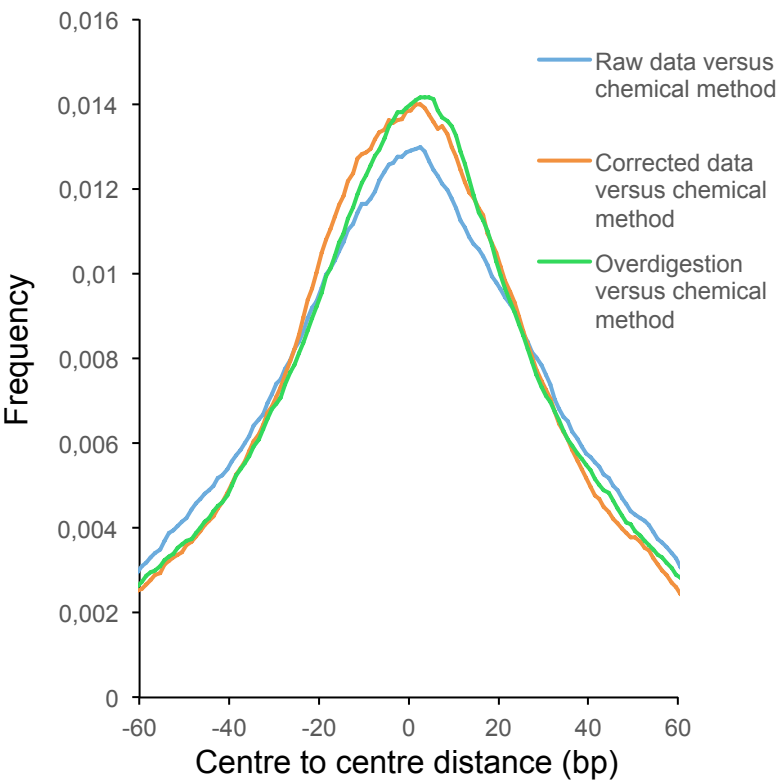

B

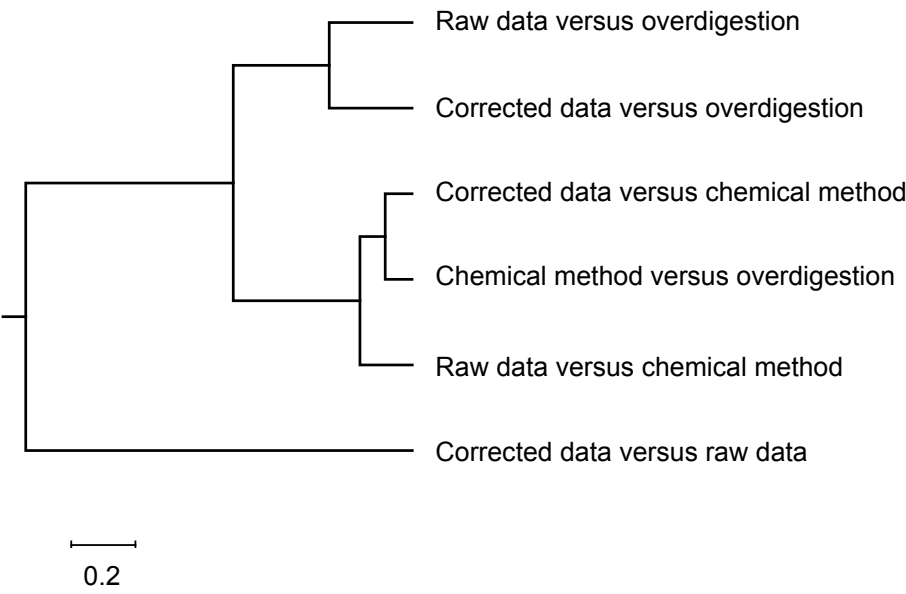

Supplement: Supplementary file 3 — Additional file 3. Comparison with chemical mapping method. A) Center-to-center distance of the nearest nucleosome in: the raw data presented here against a chemical modification-based map [31] (blue line), the corrected data against the same reference map [31] (orange line), or the chemical modification-based map against a map that was generated by extensive digestion with MNase [12]. B) Cladogram showing the distance between the different maps mentioned in A. [file 13072_2017_165_MOESM3_ESM.pdf]

## Additional file 5

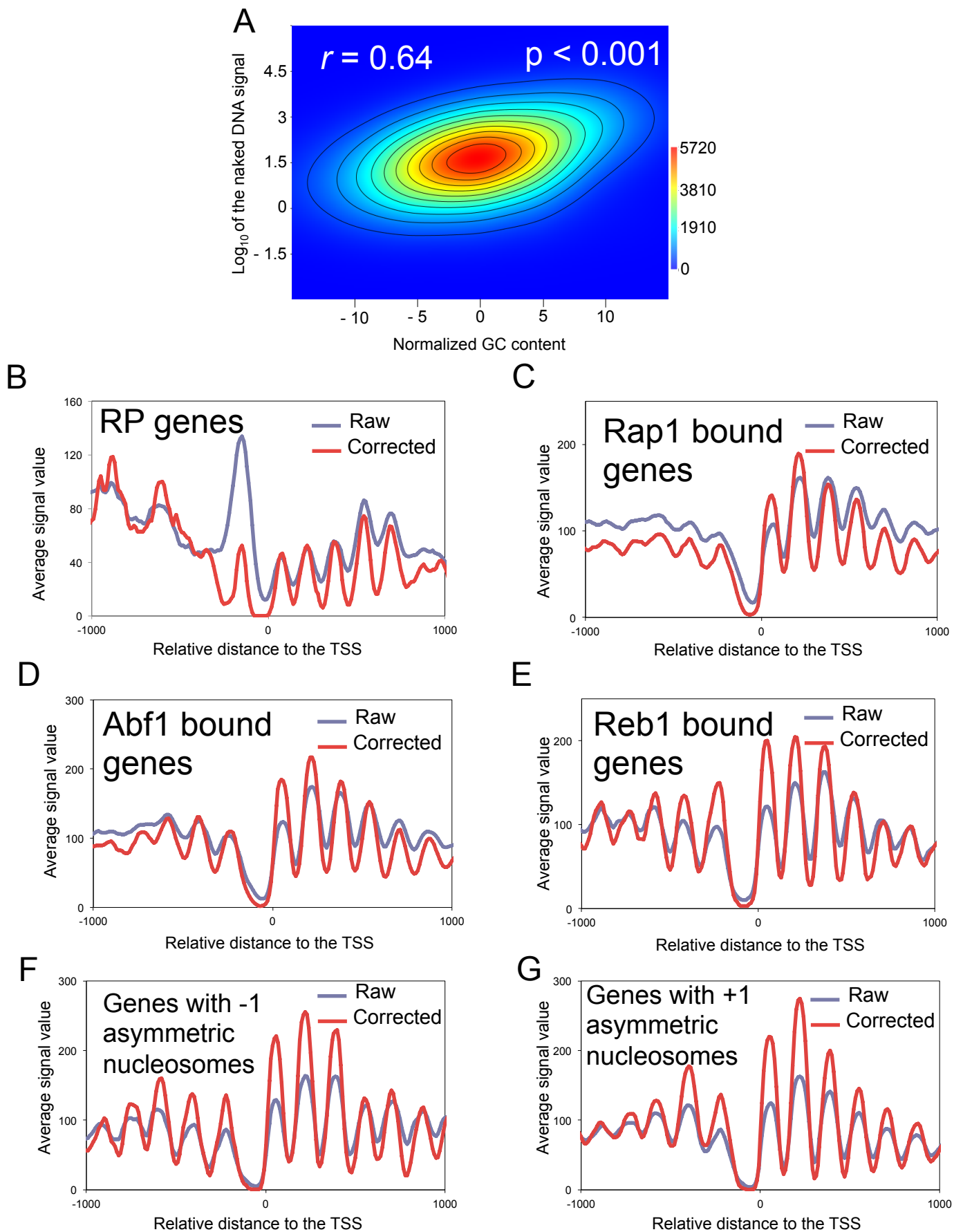

Supplement: Supplementary file 5 — Additional file 5. A metagene analysis to compare the sequencing data before and after correction in different groups of genes. A) A 2D plot to compare the log10 signal intensity in the naked DNA sample and the GC content of fragments (normalized by subtracting the genomic average). Pearson’s correlation is shown (p < 0.001). B) The metagene analysis of the region around the TSS of the ribosomal protein genes (blue before the correction, red afterward). RP genes were scaled to the same length and then aligned to their TSS. C, D, E) The metagene analysis of the 1363 genes bound by Rap1 (C), the 1311 genes bound by Abf1 (D) and the 281 genes bound by Reb1 (E), according to the DNA binding data from http://www.yeastract.com/. F, G). The metagene analysis of 115 and 392 genes that, respectively, contained a − 1 (F) or + 1 asymmetric nucleosome (G), according to the data from [44]. [file 13072_2017_165_MOESM5_ESM.pdf]

Additional file 8

YAR035w

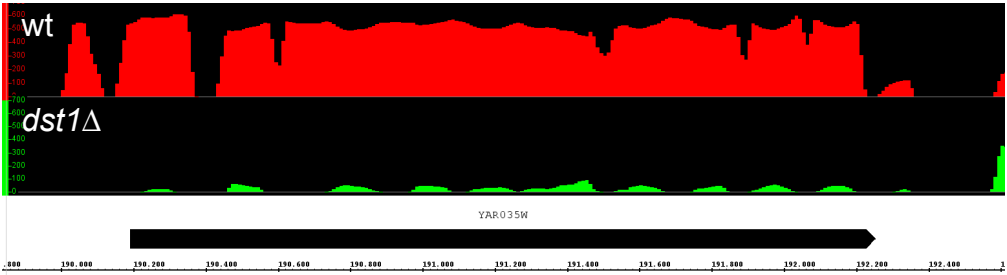

YAL021c

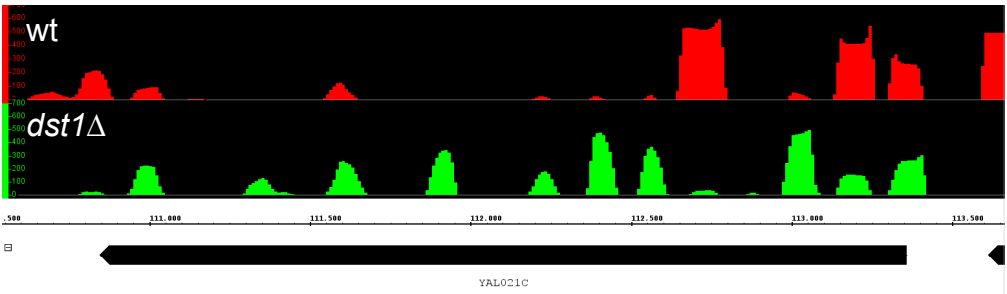

YOR348c

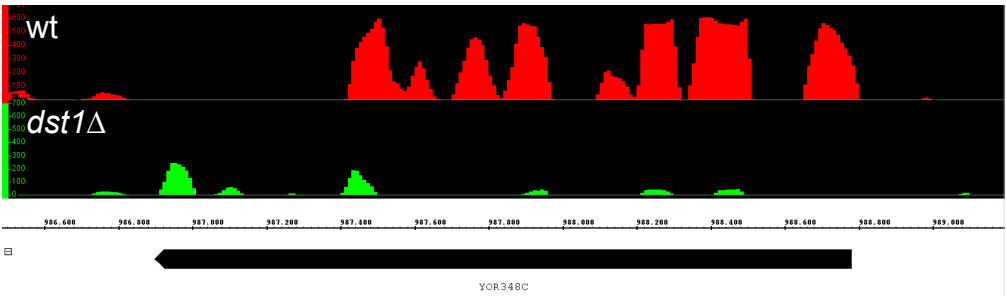

YLR438w

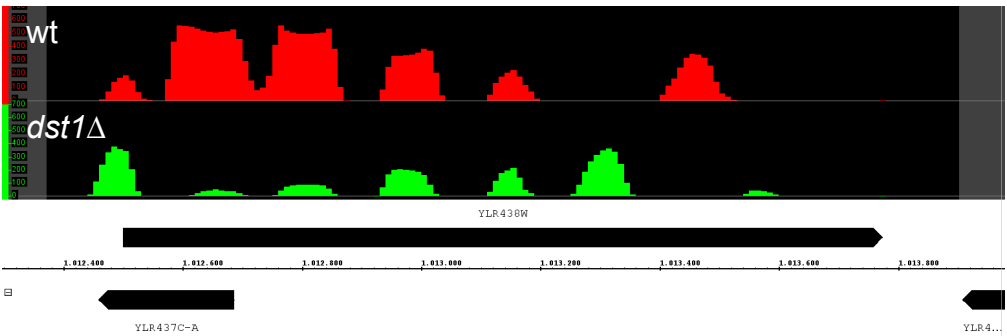

YOR328w

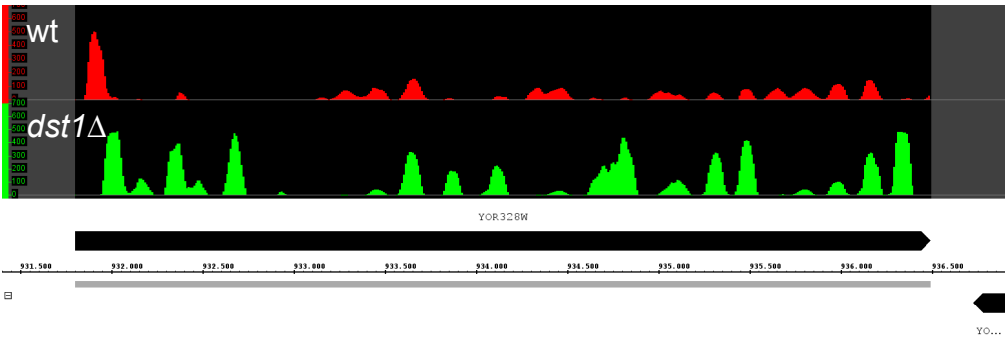

Supplement: Supplementary file 8 — Additional file 8. Nucleosome positioning of genes with significant changes between wt and dst1∆. Genes were ordered by the number of nucleosomes that changed (in occupancy or fuzziness) between the wt and dst1∆. The nucleosomal profile of the top five genes is presented. [file 13072_2017_165_MOESM8_ESM.pdf]

## Additional file 9

A

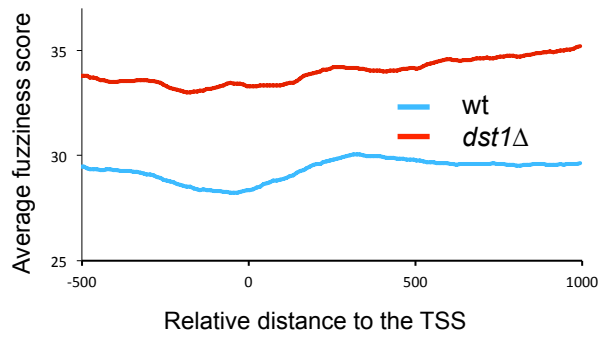

B

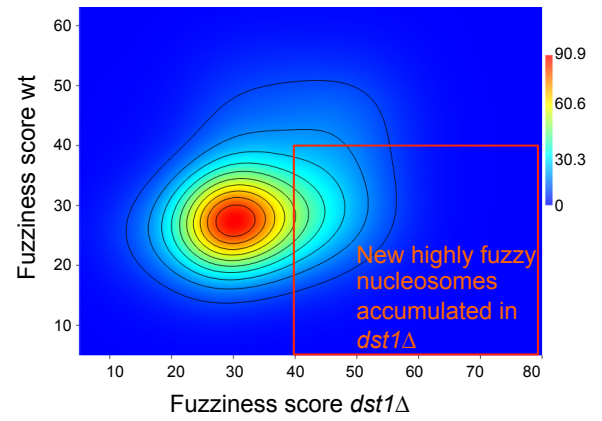

C

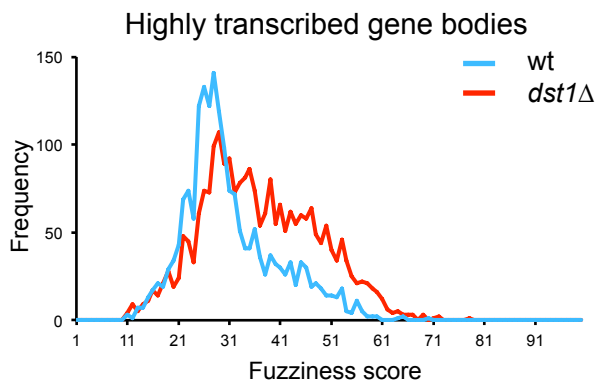

D

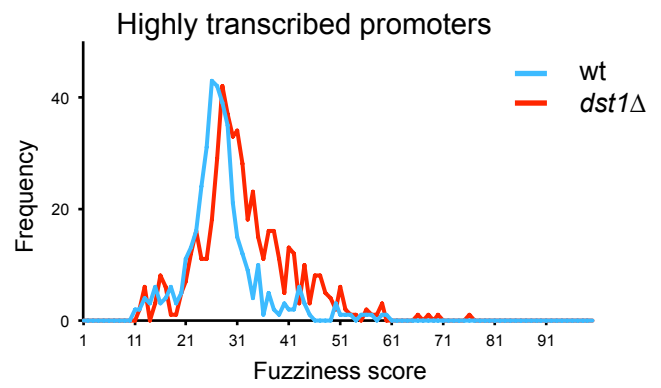

Supplement: Supplementary file 9 — Additional file 9. Nucleosome fuzziness in the wt and dst1∆. A) The metagene analysis of the fuzziness score of the wt (blue) and dst1∆ (red) nucleosomes around the TSS. Genes were scaled to the same length and then aligned to their TSS. B) The change in fuzziness score between the wt and dst1∆. Heat map of the fuzziness score of the gene body nucleosomes in the wt and dst1∆ mutant. Color represents density, which increases from blue to red. The red square highlights those nucleosomes below 40 in the wt and above > 40 in the mutant. C) The fuzziness score distribution of the nucleosomes in the gene bodies of the highly transcribed genes of the wt (blue) and dst1∆ (red). D) The fuzziness score distribution of the nucleosomes in the promoters of the highly transcribed genes of the wt (blue) and dst1∆ (red). [file 13072_2017_165_MOESM9_ESM.pdf]

## Additional file 10

A

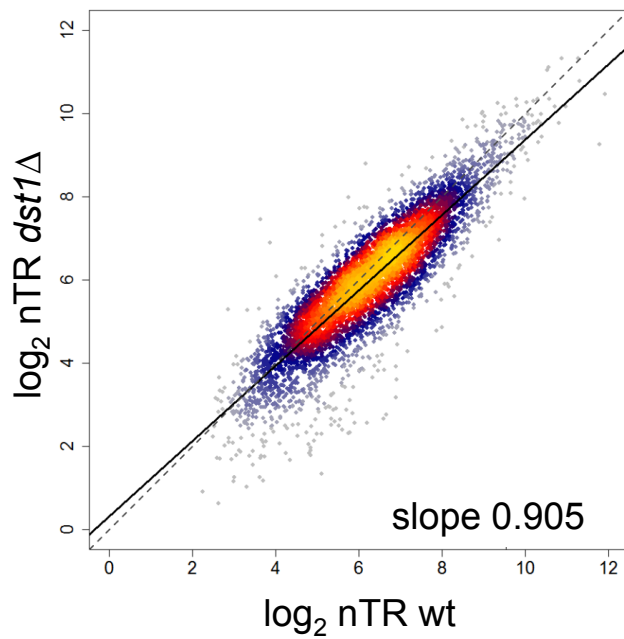

B

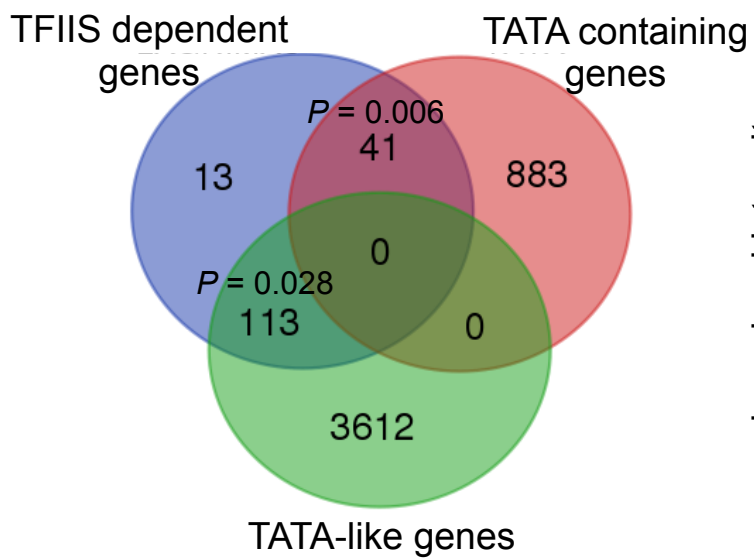

C

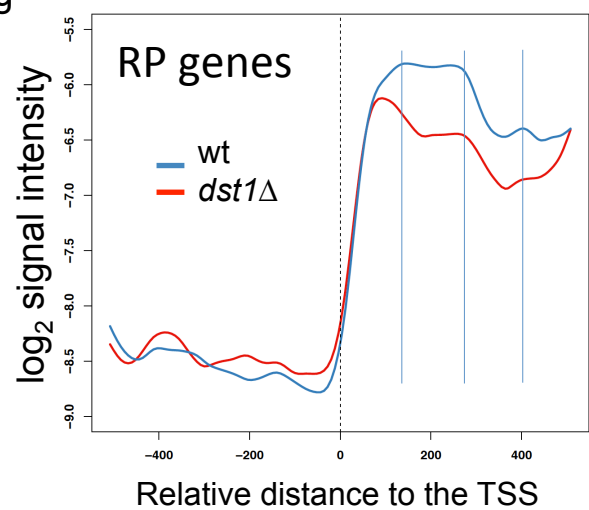

Supplement: Supplementary file 10 — Additional file 10. Effect of the absence of TFIIS on the expression of the different types of genes. A) Scatter plot of the nascent transcription rate of each gene in the wt (X-axis) and in dst1∆ (Y-axis). B) Diagram showing the relationship between the group of genes with a weaker GRO signal in dst1∆ compared to the wt (TFIIS-dependent) in the TATA-containing genes and TATA-like genes. The TATA-containing genes are overrepresented in the TFIIS-dependent genes (hypergeometric test, p = 0.006), while the TATA-like genes are under-represented (hypergeometric test, p = 0.028). C) The Bio-GRO signals of the RP genes in the wt and dst1∆. After classification, genes were aligned to their TSS. [file 13072_2017_165_MOESM10_ESM.pdf]

## Additional file 11

A

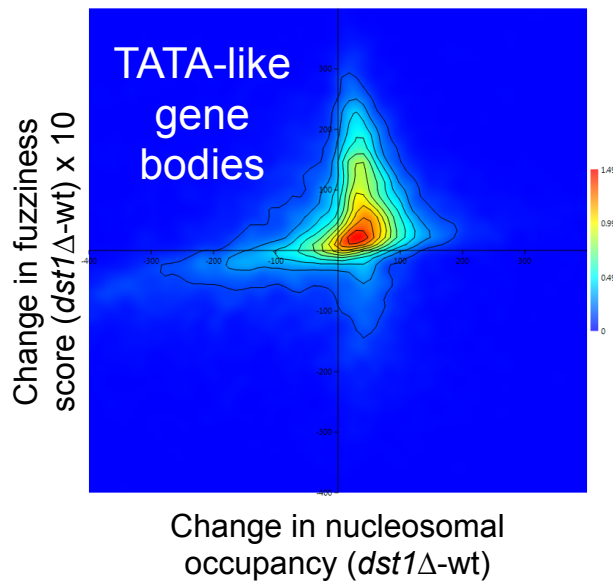

B

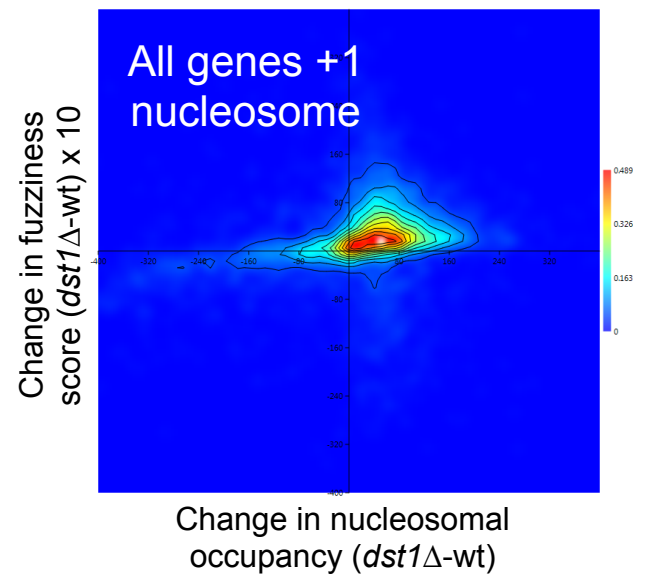

Supplement: Supplementary file 11 — Additional file 11. Occupancy-versus-fuzziness changes of the TATA-like gene bodies and + 1 nucleosomes. Heat maps of the difference between the mutant dst1∆ and the wt in fuzziness versus occupancy for the gene body nucleosomes of the TATA-like genes (A), and for the + 1 nucleosome (defined as that between the TSS and 200 bp downstream) of each gene (B). [file 13072_2017_165_MOESM11_ESM.pdf]
