# Supplementary material for: Subtracting the sequence bias from partially digested MNase-seq data reveals a general contribution of TFIIS to nucleosome positioning
Source: Epigenetics Chromatin. 2017 Dec 7;10:58. doi: 10.1186/s13072-017-0165-x (PMC5719526; doi:10.1186/s13072-017-0165-x)

Additional file 6

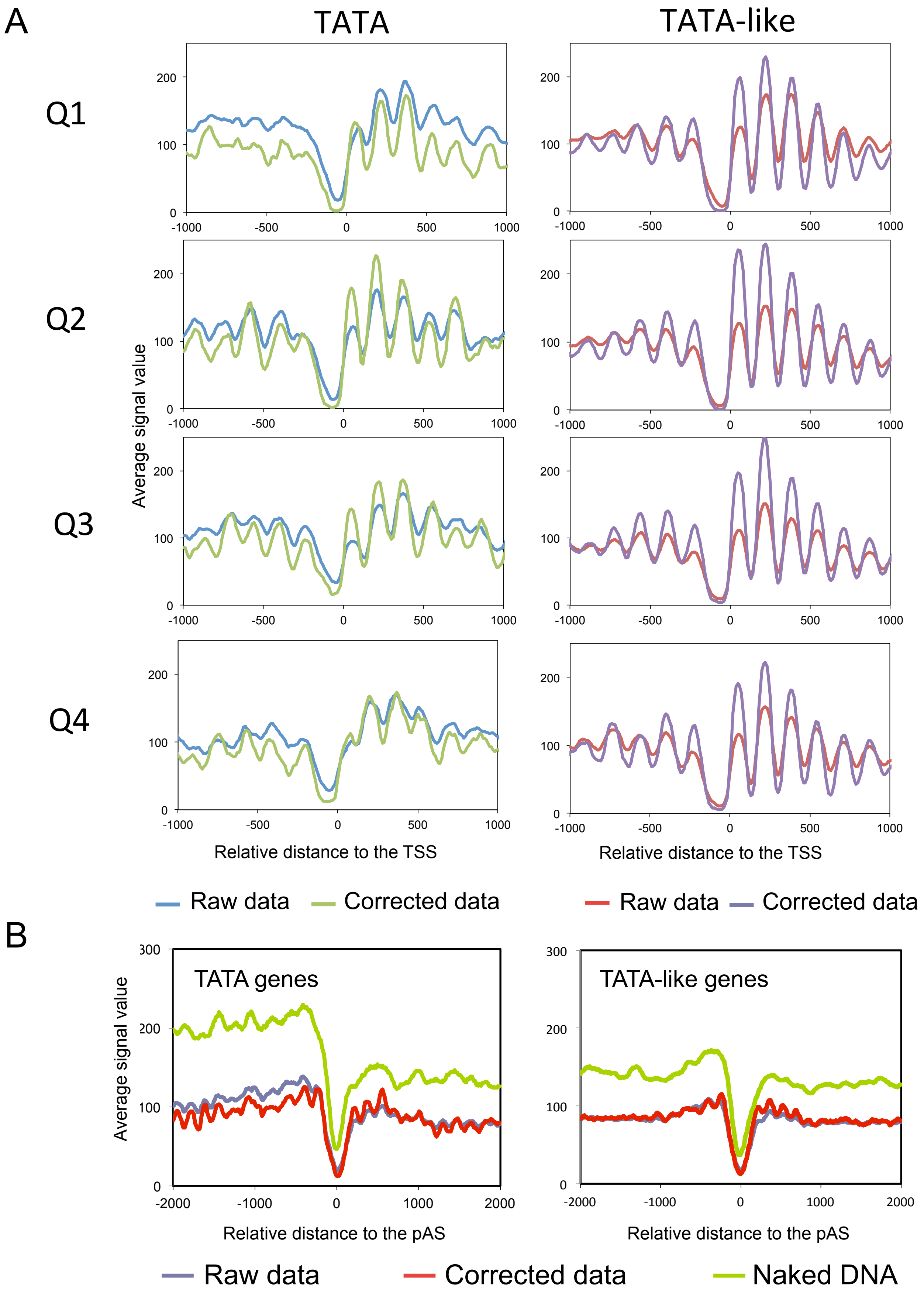

Supplement: Supplementary file 6 — Additional file 6. A metagene analysis to compare the sequencing data before and after the correction in TATA genes versus TATA-like genes A) The metagene analysis of the chromatin (blue before the correction, red afterward) and the naked DNA signals (green) around the pAS in the TATA (left panel) and TATA-like genes (right panel). Genes were scaled to the same length and then aligned to their pAS. B) Genes were divided into quartiles according to their transcription rate [45] and then further subdivided into TATA or TATA-like genes. All the resulting eight groups were scaled and aligned to their TSS. The chromatin signal before and after correction is shown. [file 13072_2017_165_MOESM6_ESM.pdf]
