# Supplementary material for: Subtracting the sequence bias from partially digested MNase-seq data reveals a general contribution of TFIIS to nucleosome positioning
Source: Epigenetics Chromatin. 2017 Dec 7;10:58. doi: 10.1186/s13072-017-0165-x (PMC5719526; doi:10.1186/s13072-017-0165-x)
Supplement: Supplementary file 7 — Additional file 7. Nucleotide composition of the sequence of the TATA and TATA-like genes. A) Frequency of each nucleotide in the TATA (red) and TATA-like genes (blue) at each position in relation to the TSS. B) The average nucleotide frequency in the promoter (− 500 to − 100) and the gene body (50–500) of the TATA and TATA-like genes. A Student’s t test was applied to compare the TATA and TATA-like genes. S indicates that the difference is significant (p < 0.001). N indicates that the difference is not significant (p > 0.001). [file 13072_2017_165_MOESM7_ESM.pdf]

Additional file 7

A

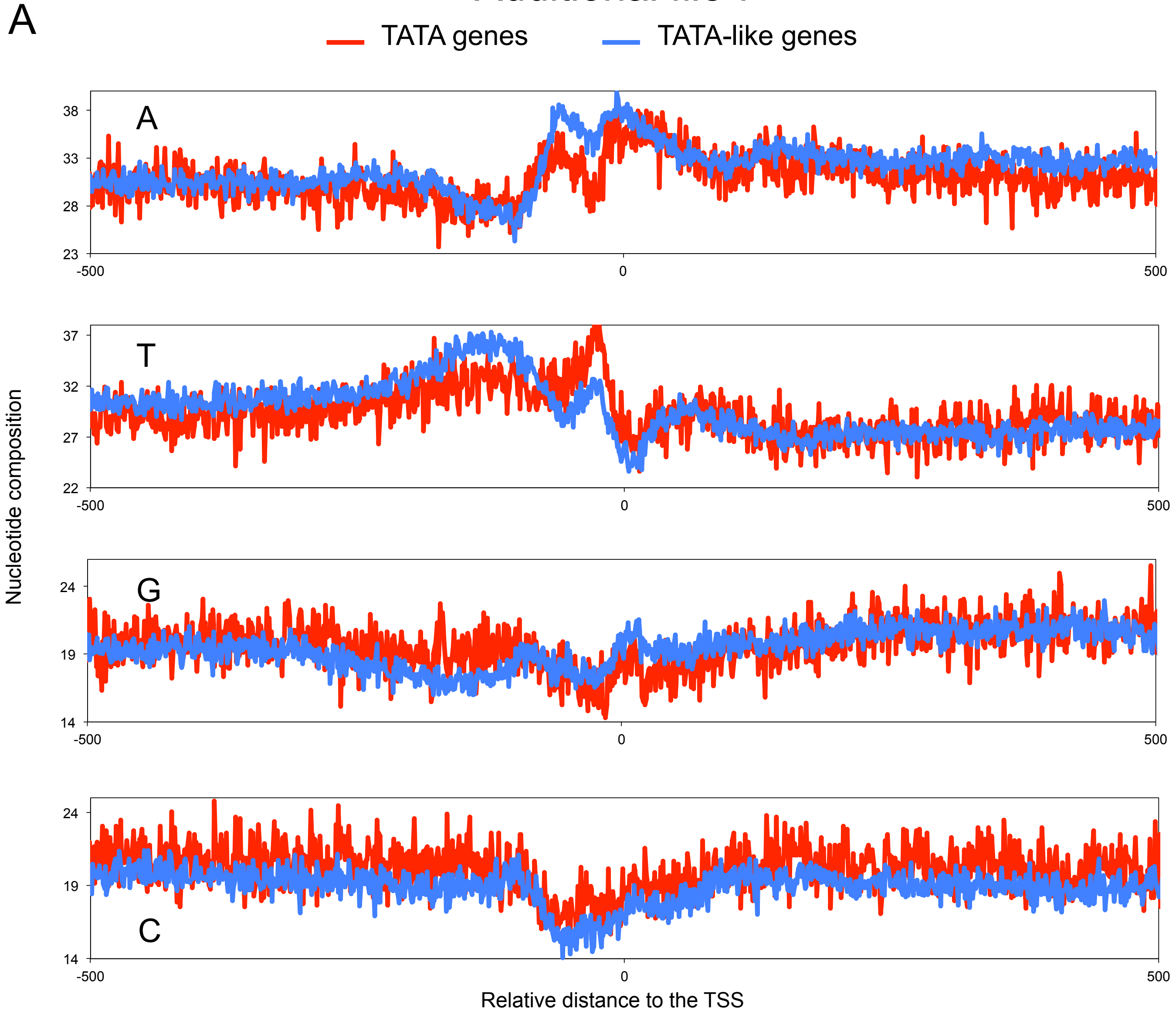

B

Promoter

	TATA	TATA-like	p<0.001
A	0.295	0.301	S
C	0.206	0.194	S
G	0.197	0.186	S
T	0.302	0.320	S

Gene body

	TATA	TATA-like	p<0.001
A	0.316	0.329	S
C	0.202	0.190	S
G	0.203	0.204	N
T	0.279	0.276	S
